# Reductive stress triggers ANAC017-mediated retrograde signaling to safeguard the endoplasmic reticulum by boosting mitochondrial respiratory capacity

**DOI:** 10.1093/plcell/koac017

**Published:** 2022-01-25

**Authors:** Philippe Fuchs, Finja Bohle, Sophie Lichtenauer, José Manuel Ugalde, Elias Feitosa Araujo, Berivan Mansuroglu, Cristina Ruberti, Stephan Wagner, Stefanie J Müller-Schüssele, Andreas J Meyer, Markus Schwarzländer

**Affiliations:** Institute of Plant Biology and Biotechnology (IBBP), Westfälische Wilhelms-Universität Münster, D-48143 Münster, Germany; Institute of Crop Science and Resource Conservation (INRES), Rheinische Friedrich-Wilhelms-Universität Bonn, D-53113 Bonn, Germany; Institute of Crop Science and Resource Conservation (INRES), Rheinische Friedrich-Wilhelms-Universität Bonn, D-53113 Bonn, Germany; Institute of Plant Biology and Biotechnology (IBBP), Westfälische Wilhelms-Universität Münster, D-48143 Münster, Germany; Institute of Crop Science and Resource Conservation (INRES), Rheinische Friedrich-Wilhelms-Universität Bonn, D-53113 Bonn, Germany; Institute of Plant Biology and Biotechnology (IBBP), Westfälische Wilhelms-Universität Münster, D-48143 Münster, Germany; Institute of Crop Science and Resource Conservation (INRES), Rheinische Friedrich-Wilhelms-Universität Bonn, D-53113 Bonn, Germany; Institute of Plant Biology and Biotechnology (IBBP), Westfälische Wilhelms-Universität Münster, D-48143 Münster, Germany; Institute of Plant Biology and Biotechnology (IBBP), Westfälische Wilhelms-Universität Münster, D-48143 Münster, Germany; Institute of Crop Science and Resource Conservation (INRES), Rheinische Friedrich-Wilhelms-Universität Bonn, D-53113 Bonn, Germany; Institute of Crop Science and Resource Conservation (INRES), Rheinische Friedrich-Wilhelms-Universität Bonn, D-53113 Bonn, Germany; Institute of Crop Science and Resource Conservation (INRES), Rheinische Friedrich-Wilhelms-Universität Bonn, D-53113 Bonn, Germany; Institute of Plant Biology and Biotechnology (IBBP), Westfälische Wilhelms-Universität Münster, D-48143 Münster, Germany; Institute of Crop Science and Resource Conservation (INRES), Rheinische Friedrich-Wilhelms-Universität Bonn, D-53113 Bonn, Germany

## Abstract

Redox processes are at the heart of universal life processes, such as metabolism, signaling, or folding of secreted proteins. Redox landscapes differ between cell compartments and are strictly controlled to tolerate changing conditions and to avoid cell dysfunction. While a sophisticated antioxidant network counteracts oxidative stress, our understanding of reductive stress responses remains fragmentary. Here, we observed root growth impairment in *Arabidopsis thaliana* mutants of mitochondrial *alternative oxidase 1a* (*aox1a*) in response to the model thiol reductant dithiothreitol (DTT). Mutants of mitochondrial *uncoupling protein 1* (*ucp1*) displayed a similar phenotype indicating that impaired respiratory flexibility led to hypersensitivity. Endoplasmic reticulum (ER) stress was enhanced in the mitochondrial mutants and limiting ER oxidoreductin capacity in the *aox1a* background led to synergistic root growth impairment by DTT, indicating that mitochondrial respiration alleviates reductive ER stress. The observations that DTT triggered nicotinamide adenine dinucleotide (NAD) reduction in vivo and that the presence of thiols led to electron transport chain activity in isolated mitochondria offer a biochemical framework of mitochondrion-mediated alleviation of thiol-mediated reductive stress. Ablation of transcription factor Arabidopsis NAC domain-containing protein17 (ANAC017) impaired the induction of *AOX1a* expression by DTT and led to DTT hypersensitivity, revealing that reductive stress tolerance is achieved by adjusting mitochondrial respiratory capacity via retrograde signaling. Our data reveal an unexpected role for mitochondrial respiratory flexibility and retrograde signaling in reductive stress tolerance involving inter-organelle redox crosstalk.

## Teaser

Enhancement of mitochondrial respiratory capacity and its control by retrograde signaling aid thiol oxidation and serve reductive ER stress tolerance in Arabidopsis.

IN A NUTSHELL **Background:** A strict division into specialized compartments, such as the cytosol, the chloroplasts, the mitochondria, or the vacuole, is a principle that underpins the versatility of plant cells. For the different compartments to work in concert, they must each maintain their characteristic environment which needs to be defended against deviations. While the cytosol and the mitochondrial matrix require the thiol groups of their proteins and metabolites to be reduced, oxidative folding of secreted proteins in the endoplasmic reticulum (ER) requires conditions that favor oxidation to generate disulfide bridges. As a consequence, the ER is particularly susceptible to deviations toward reduction by intake of thiols. To combat reductive stress, the ER requires robust response mechanisms.

**Question:** We made the serendipitous observation that *Arabidopsis thaliana* mutants with defects in their mitochondrial respiratory machinery were hypersensitive to reductive ER stress. We hence asked the question if and how mitochondrial respiration may support ER redox homeostasis.

**Finding:** Reductive ER stress can be induced by the model thiol reductant dithiothreitol (DTT). DTT impairs root growth in Arabidopsis seedlings and triggers an emergency program called the Unfolded Protein Response (UPR_ER_). We found that the ER stress responses were more severe when mechanisms that allow flexible tuning of respiration in the mitochondria were impaired, indicating that the oxidation capacity of the mitochondrial electron transport chain can support ER function. To understand the underlying mechanisms, we assessed the subcellular redox dynamics of glutathione and the nicotinamide adenine dinucleotide (NAD) pool in living seedlings using fluorescent protein biosensors. We found that thiol-mediated reductive stress can affect NAD redox metabolism and that mitochondria can act as sink for the excess reductant. The data pinpoint a new role for the mitochondria in safeguarding the ER.

**Next steps:** The metabolic mechanism that links mitochondrial respiration to thiol oxidation deserves further investigation. The role that the physical association between mitochondria and the ER in the cell plays may be of interest in that context. Further, it needs testing to what extent the model can be generalized to naturally occurring conditions that induce ER stress, as well as to non-plant systems.

## Introduction

Redox biochemistry is the driver behind many life processes, including photosynthesis, respiration, and folding of proteins for secretion (Buchanan and Balmer, 2005). Any oxidation must be stoichiometrically linked to a reduction, which poses a fundamental constraint to cells. Deviation from redox balance bears the risk of a series of malfunctions, such as metabolic inhibition, the excessive production of reactive oxygen species (ROS), or misfolding of proteins (Waszczak et al., 2018; Bhattarai et al., 2021; Meyer et al., 2021). As an adaptive strategy, an elaborate network of redox compounds and proteins ensures buffering and active redox regulation. The mitochondrial electron transport chain (mETC) is central to that network, as it equips the cell with a high capacity electron sink using molecular oxygen as final acceptor. As an electron acceptor, the mETC provides flexibility to compatible redox reactions (Braun, 2020). Any limitation in the mETC capacity, however, can lead to a reduction of the cellular nicotinamide adenine dinucleotide (NAD) pools with knockon effects across the metabolic network and also to oxidative stress through single electron transfer by redox centers of the mETC generating ROS. Plants can partially circumvent limitations in mETC flux through a high degree of flexibility in respiratory coupling, as mediated by alternative NAD(P)H dehydrogenases (NDH), alternative oxidases (AOX), and uncoupling proteins (UCP; Borecký and Vercesi, 2005; Rasmusson et al., 2008). Those systems have in common that they allow more electrons to be transported to oxygen per ATP generated, tuning the mETC between being efficient in energy conservation by phosphorylation or rather having peak capacity for the safe disposal of cellular reducing equivalents.

A key role of respiratory flexibility as mediated by UCP1 has been attributed to photorespiratory metabolism, but also drought and salt stress responses (Sweetlove et al., 2006; Begcy et al., 2011; recently reviewed by Barreto et al., 2020). Loss of AOX1a has been reported to increase susceptibility of leaves to combined drought and light stress and other conditions challenging the energy and/or carbon balance in the photosynthetic cell, in agreement with a role in providing an electron sink to avoid photoinhibition (Giraud et al., 2008; Smith et al., 2009; Kühn et al., 2015; Shapiguzov et al., 2019; Alber and Vanlerberghe, 2021)*. AOX1a* has been extensively studied as a core stress response gene in Arabidopsis, emphasizing the general need for avoiding metabolic over-reduction under a variety of stress conditions (Escobar et al., 2006; Giraud et al., 2008; Smith et al., 2009). *AOX1a* expression responds strongly to mETC inhibition and is a core component of the *mitochondrial dysfunction stimulon* (MDS; also named *mitochondrial dysfunction regulon*; Vanderauwera et al., 2012; De Clercq et al., 2013; Shapiguzov et al., 2020), based on which it has been used as a model to study *mitochondrial retrograde regulation* (MRR). Several other MDS members have been identified through a genetic screen based on the response of *AOX1a* to the complex III inhibitor antimycin A (AA) (Giraud et al., 2009; Van Aken et al., 2013; Ng et al., 2013a, 2013b). A key mechanism of *AOX1a* induction includes cleavage of endoplasmic reticulum (ER) membrane-bound transcription factors Arabidopsis NAC domain-containing protein17 (ANAC017) and ANAC013, followed by their release and translocation to the nucleus (De Clercq et al., 2013; Ng et al., 2013a). Signaling from other organelles and hormonal regulation further affect *AOX1a* expression, giving rise to the highly integrated regulation of AOX1a function and the possibility of fine-tuning at multiple levels (Shapiguzov et al., 2019, and recently reviewed by Selinski et al., 2018; Wang et al., 2020).

Even though NAC-based MRR via the ER may appear counterintuitive at first, mitochondria and the ER are closely associated in the plant cell and interact physically (Jaipargas et al., 2015; Müller and Reski, 2015; White et al., 2020). Hence, local signaling interaction between both organelles is not required when including a cell biological perspective. Interaction and exchange between mitochondria and the ER have been demonstrated in mammalian systems, including direct lipid and Ca^2+^ transfer via dedicated interaction complexes (Aaltonen et al., 2016; Gomez-Suaga et al., 2017). Similar interactions and exchanges are likely to also operate in plants but remain to be investigated (Michaud et al., 2016; Michaud and Jouhet, 2019).

One of the key functions of the ER is the processing of proteins for secretion. This includes oxidative protein folding which requires oxidation of cysteine (Cys) residues to form specific disulfides that stabilize the structure of the mature protein (Radzinski et al., 2021). While Cys residues of cytosolic proteins are typically maintained in a reduced state through highly reducing thiol-based redox systems, disulfide formation is actively driven in the ER through ER oxidoreductins (ERO) which exist as two isoforms in Arabidopsis that transfer electrons to molecular oxygen, generating hydrogen peroxide (H_2_O_2_) (Aller and Meyer, 2013; Fan et al., 2019; Meyer et al., 2019). Insufficient oxidation rates in this system can be triggered by constraints in electron removal under hypoxia, for example, during water logging, or by peak rates of protein secretion as induced by developmental needs, defense, or other stress responses, for example, cell division, tip growth of root hairs, or pathogen defense. In consequence, lack of oxidative power for protein folding leads to reductive ER stress and can induce the Unfolded Protein Response (UPR_ER_). The UPR_ER_ has been studied extensively across eukaryotes and a mechanistic understanding of the conserved UPR_ER_ pathway and its components has been gained. Model stimuli for UPR_ER_ are dithiothreitol (DTT, to induce thiol-based reductive stress) and tunicamycin (TM, blocking *N*-glycosylation ; Chen and Brandizzi, 2012; Deng et al., 2013; Maity et al., 2016; Ruberti and Brandizzi, 2018). The cell physiological context by which the ER may be safeguarded from reductive stress remains poorly defined in any organism, however.

Here, we observed that Arabidopsis mutant lines with limited ability to boost their mitochondrial respiratory capacity are hypersensitive to thiol-based reductive stress. Since the plants showed characteristic ER stress phenotypes, we delineated how mitochondrial respiration can alleviate thiol-based reductive stress in the ER. We combined reverse genetics, chemical biology, stress phenotyping, gene expression analyses of signaling responses, and fluorescent protein-based biosensing of redox dynamics and mitochondrial function to pinpoint the relationship between mitochondrial respiration and ER stress. Our data reveal a role for mitochondrial respiratory flexibility and MRR in safeguarding ER function.

## Results

### AOX1a is required for root growth under thiol-mediated reductive challenge

**Figure 1:**
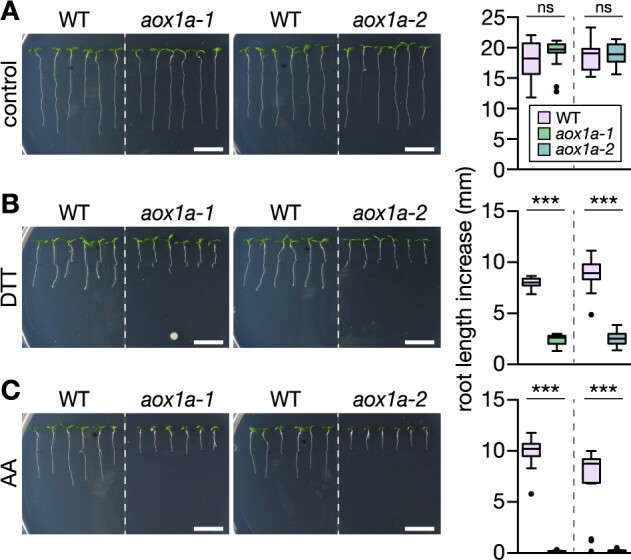
Arabidopsis *aox1a* seedlings show drastically impaired root growth at DTT exposure. Primary root length increase of WT Col-0 and *aox1a-1* and *aox1a-2* seedlings. Seedlings were grown vertically on half-strength MS agar plates for 4 days, then transferred to fresh plates supplemented with the treatment chemicals. A, Control; B, 800 µM DTT; C, 20 µM antimycin A (AA). (Left) representative images of seedlings 4 days after transfer. Scale bars: 10 mm. (Right) Root length increase measured 4 days after transfer. (A) *N* = 11–18, (B) *N* = 16–18, (C) *N* = 17–18. Boxplot: first and third quartiles with median and Tukey’s whiskers. Differences were tested after log-transformation of data to establish normal distribution by one-way ANOVA with Bonferroni’s multiple comparisons test (^ns^*P* > 0.05, ***P < 0.001). *P*-values: Supplemental Data Set 1.

In the course of investigating the role of abiotic factors, ROS and redox regulation in MRR, we made the serendipitous observation that root growth of Arabidopsis lines lacking mitochondrial AOX1a (*aox1a-1* and *aox1a-2*; Giraud et al., 2008; Kühn et al., 2015) was strongly sensitized to the thiol-based reducing compound DTT when added to the growth medium (Figure 1, A and B). DTT is readily oxidized when dissolved in aqueous solutions, especially at elevated temperatures (Stevens et al., 1983), leading to variations of reduced, efficacious DTT in the media of plates between experiments. Hence, preparation of treatment media was strictly standardized (see “Materials and Methods” section) and comparisons between genotypes were performed through side-by-side comparisons on the same medium plates. To consider the potential variability in effective concentration of the treatment, DTT concentrations between 400 and 800 µM were routinely assessed (Figure 1; Supplemental Figure S1). Despite remaining variability in the overall effectiveness of the DTT treatment between experiments, the *aox1a* phenotype was strictly reproducible, as apparent by stronger impairment of root growth in the *aox1a* lines compared to their corresponding wild-type (WT) control in the presence of DTT in the high micromolar range. No obvious differential behavior was observed in green tissues between the genetic backgrounds, which was not specifically investigated here. Instead, we focused our further quantitative assessment on roots. Root growth was almost completely suppressed in the *aox1a* backgrounds by DTT, as induced by 20 µM of the complex III inhibitor AA (Figure 1C). Complex III inhibition can be tolerated in plants due to the electron bypass via AOX, but not when AOX activity is diminished through absence of AOX1a (Strodtkötter et al., 2009). None of the other stress treatments that we tested elicited similar phenotypic differences between WT and *aox1a* seedlings (exemplified in Supplemental Figure S2), suggesting a functional role of AOX1a at exposure of seedlings to high micromolar concentrations of DTT. While impaired *AOX1a* expression is known to interfere with photosynthetic physiology in green tissues, rendering plants hypersensitive to high light and drought (Giraud et al., 2008; Wang and Vanlerberghe, 2013; Kaye et al., 2019), the root growth phenotype suggested a function beyond photosynthetic tissues in tolerance to thiol-based reductive stress.

### Defining the effect of DTT on ER stress in roots

DTT has been widely used to induce ER stress across eukaryotic models. As a small membrane-permeable thiol compound, it reduces endogenous disulfide bonds through thiol-disulfide exchange. Those are the exception in most intracellular compartments but are common in the compartments of the secretory pathway, since disulfides are actively generated through thiol oxidation at high rate in the ER. As a result DTT preferentially interferes with maturation of proteins in the secretory pathway and induces proteotoxic ER stress (Martínez and Chrispeels, 2003; Iwata et al., 2008). To define the effect that DTT has on Arabidopsis seedling roots under the experimental conditions used, we assessed (1) Cys redox status in the ER lumen as compared to other major subcellular compartments in vivo, (2) induction of the UPR_ER_, and (3) root growth in a mutant of ERO, the terminal thiol oxidase in the ER.

**Figure 2:**
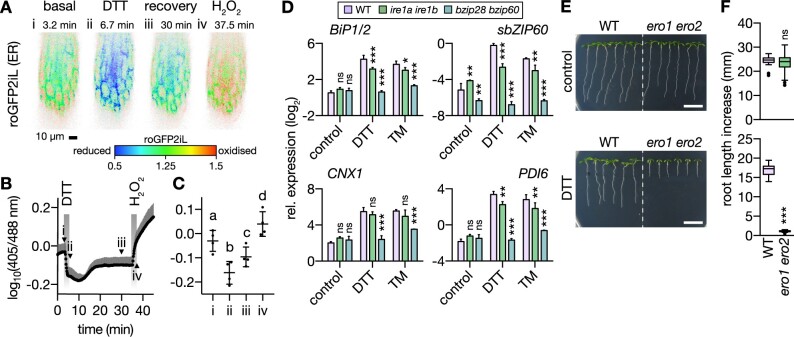
DTT causes reductive ER stress in roots of Arabidopsis seedlings. A–C, Four-day-old Arabidopsis seedling roots expressing ER lumen-targeted Grx1-roGFP2iL were perfused with control medium and exposed to 5 mM DTT or 10 mM H_2_O_2_, respectively. A, Ratiometric images of one root section during a representative time course experiment. High 405/488-nm excitation ratios indicate a less negative *E*_GSH_ (red); low ratios indicate a more negative *E*_GSH_ (blue). Images from four time points are shown and marked i–iv as indicated in (B). B, Corresponding time series data. *N* = 4. Mean + sd. Arrow heads indicate time points of images i–iv in (A). C, Indicated time points from (A) and (B). Mean ± sd. *N* *=* 4. Different letters indicate significant differences (one-way ANOVA with Tukey’s multiple comparisons test; *P* < 0.01). D, ER stress marker transcript quantification by RT-qPCR from roots of 12-day-old seedlings submerged in treatment solutions without (control) or with 2 mM DTT or 4.3 μg mL^−1^ TM for 2.5 h. Lines: WT Col-0, *ire1a ire1b*, *bzip28 bzip60*. Transcripts: *BiP1/2*, *sbZIP60*, *CNX1*, or *PDI6*. Expression level is shown relative to reference transcript *TIP41*. Mean + sd. *N* = 3 plates, with pooled roots from 18 to 20 seedlings each. Note that the stronger suppression in *bzip28 bzip60* than in *ire1a ire1b* correlated with root growth suppression in the mutants in the presence of DTT (Deng et al., 2013). Differences were tested by two-way ANOVA with Dunnett’s multiple comparisons test (^ns^*P* > 0.05, *P < 0.05, **P < 0.01, ***P < 0.001). E, Representative images of WT and *ero1 ero2* seedlings 4 days after transfer to new plates. Plants were grown vertically on half-strength MS agar plates for 4 days, then transferred to fresh plates without (control) or with 400 µM DTT. Scale bars: 10 mm. F, Primary root growth within 4 days after transfer on new plates. (Top) Control, *N* = 19–24. (Bottom) DTT, *N* = 18. Boxplot: first and third quartiles with median and Tukey’s whiskers. Differences were tested after log-transformation of data to establish normal distribution by one-tailed *t*-test (^ns^*P* > 0.05, ***P < 0.001). *P*-values: Supplemental Data Set 2.

(1) First, we used the fluorescent protein redox sensor roGFP2 to measure the relative effect of DTT treatments in the high micromolar and low millimolar range on the redox potential of glutathione (*E*_GSH_; as the major thiol metabolite and DTT target) in five subcellular compartments of Arabidopsis seedlings, that is, the cytosol, peroxisomes, plastid stroma, mitochondrial matrix, and the ER lumen. The *E*_GSH_ in the ER was the most oxidizing and showed high responsiveness to DTT. The other cell compartments maintained highly reducing glutathione (GSH) pools that were hardly affected by the DTT treatments (Supplemental Figure S3A). Next, we recorded the in vivo DTT response in roots using ER-localized Grx1-roGFP2iL, which has been engineered for high sensitivity in oxidizing environments (Aller et al., 2013; Schwarzländer et al., 2016; Müller-Schüssele et al., 2021), and confirmed *E*_GSH_ reduction in the ER lumen (Figure 2, A–C). Removal of DTT resulted in rapid recovery, reflecting the endogenous potential of the ER to re-establish its redox balance. Reduction was observable down to a DTT concentration of 1 µM (Supplemental Figure S3, B and C). (2) To assess the effect of DTT on ER function, we measured UPR_ER_ induction in the seedling roots using the UPR_ER_ marker transcripts, *BINDING PROTEIN 1* and 2 (*BiP1/2*), *spliced* *BASIC LEUCINE ZIPPER 60* *(sbZIP60)*, *CALNEXIN 1* (*CNX1*), and *PROTEIN DISULFIDE ISOMERASE 6* (*PDI6*) using acute exposure with 2 mM DTT for 2.5 h (Figure 2D). They were strongly induced and their induction was UPR_ER_-specific since it was impaired in the UPR_ER_ signaling backgrounds *inositol requiring enzyme 1a* and *b* (*ire1a ire1b*) and *basic leucine zipper 28* and *60* (*bzip28 bzip60*; Chen and Brandizzi, 2012; Deng et al., 2013). (3) Since impairment of root growth is a hallmark of ER stress in plants (Deng et al., 2013; Lai et al., 2018), we tested the effect of lowering the endogenous cellular oxidation capacity in the ER using a line compromised in both ERO isoforms (*ero1 ero2*; Ugalde et al., 2021a). The *ero1 ero2* seedlings showed arrested root growth in the presence of DTT, while that of WT seedlings decreased only moderately (−31%). Root growth of *ero1 ero2* was indistinguishable from WT in the absence of DTT (Figure 2, E and F). Taken together these observations validate that DTT induces reduction of disulfides in the ER and activates the UPR_ER_. Perturbing the endogenous oxidation machinery of the ER genetically sensitizes the root to severe growth impairment at DTT exposure, which is not apparent under nonstressed conditions. We next exploited this system to unravel the potential connection of AOX1a and mitochondrial respiration with ER stress.

### *ucp1* shows similar sensitivity to thiol-mediated reductive stress as *aox1a* suggesting respiratory flexibility is required for tolerance

**Figure 3:**
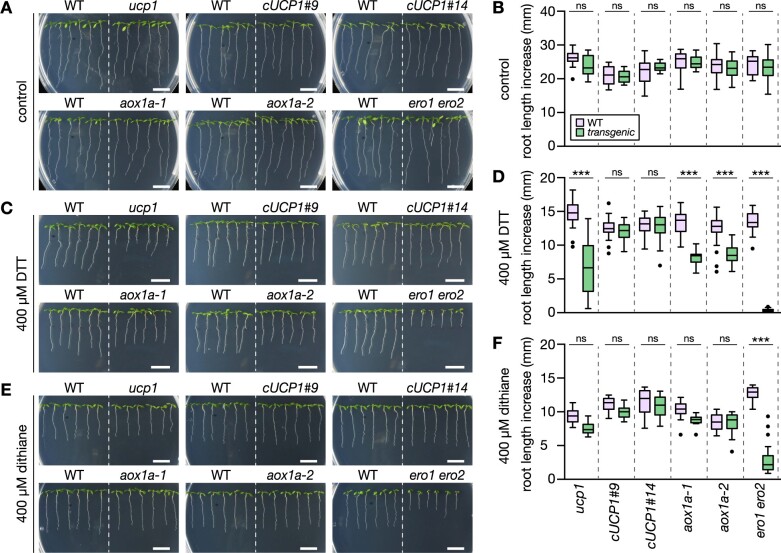
Arabidopsis *aox1a* and *ucp1* seedlings show similar impairment of root growth as caused by the DTT thiol. Primary root length increase of WT Col-0 and transgenic seedlings (*ucp1*, c*UCP1#9* and *#14*: *ucp1* complemented with UCP1 under the control of the native promoter, *aox1a* and *ero1 ero2*). Seedlings were grown vertically on half-strength MS agar plates for 4 days, then transferred to fresh plates supplemented with the treatment chemicals. A, Control; C, DTT; E, dithiane. Representative images of seedlings 4 days (A and C) or 3 days (E) after transfer. Scale bars: 10 mm. B, D, and F, Root length was measured 4 days (B and D) or 3 days (F) after transfer. (B) *N* = 17–18, (D) *N* = 28–36, (F) *N* = 18–24. Boxplot: first and third quartiles with median and Tukey’s whiskers. Differences were tested after log-transformation of data to establish normal distribution by one-way ANOVA with Bonferroni’s multiple comparisons test (^ns^*P* > 0.05, ***P < 0.001). *P*-values: Supplemental Data Set 3. Note that *ero1 ero2* root growth was suppressed also by dithiane (−77%). Dithiane does not possess reduced thiol groups but can pass cellular membranes. Once inside the cell it may be reduced by the cytoplasmic redox machineries to form DTT. DTT can then enter the ER, which is a likely cause of reductive stress in the ER, even in the absence of external net electron supply. Dithiane-mediated re-distribution of endogenous electrons between cell compartments leading to ER reduction is also supported by Supplemental Figure S3, D–G.

We explored whether the function of AOX1a in providing respiratory flexibility and increased mitochondrial electron transport activity by uncoupling contributes to tolerance to thiol-based reductive stress. We used a mutant line of the UCP1, *ucp1-1* and two complemented lines, *cUCP1* #9 and #14 (Sweetlove et al., 2006), following the rationale that the same net-effect in uncoupling and increasing electron transport activity can be achieved by either bypassing proton pumping (via AOX) or by dissipating the proton-gradient downstream of electron transport (via UCP). Transfer of seedlings to DTT plates inhibited primary root length increase of *ucp1* and *aox1a* seedlings (Figure 3, A–D). While root elongation of *ucp1* was reduced by 55% relative to WT, root elongation in *cUCP1* #9 and #14 was indistinguishable from WT seedlings. The effect of DTT on *ucp1* root elongation was even stronger than on the *aox1a* lines, which showed a decrease by 38% (*aox1a-1*) and 31% (*aox1a-2*) compared to WT. Root elongation in the *ero1 ero2* control was completely abolished. Oxidized DTT (trans-4,5-dihydroxy-1,2-dithiane; dithiane) did not impair root elongation of *ucp1* and *aox1a* to any further extent than of WT seedlings, demonstrating that the added thiols elicited the phenotypic differences (Figure 3, E and F). Over-proportionally strong root growth impairment in *ucp1* and *aox1a* seedlings was reproducible at different DTT concentrations, affecting both primary and lateral root growth (Supplemental Figure S4). Those data indicate that different mechanisms of respiratory uncoupling and increased electron transport flexibility can alleviate reductive stress, and their specific involvement depends on stress severity.

### The response of the ER to reductive stress depends on mitochondrial respiratory flexibility

**Figure 4:**
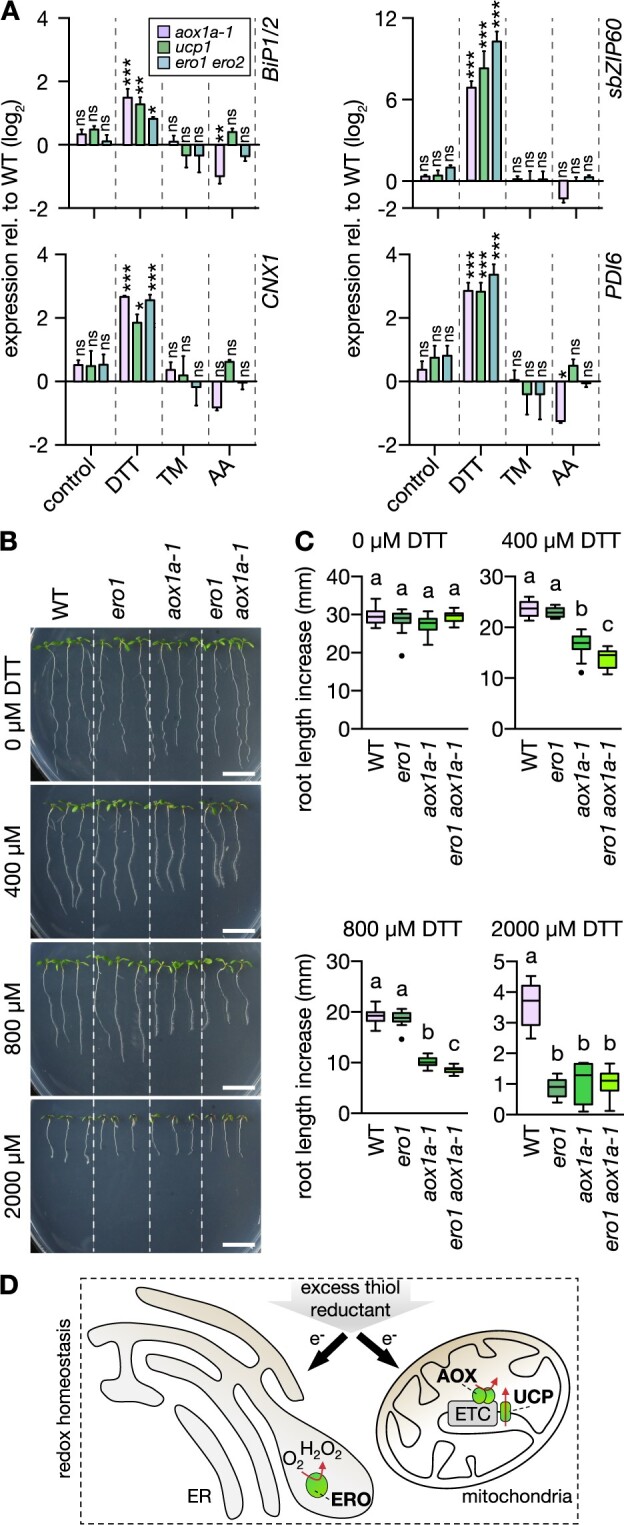
*aox1a* and *ucp1* seedlings suffer from more severe ER stress at DTT exposure. A, ER stress marker transcript quantification by RT-qPCR from roots of 12-day-old seedlings submerged in treatment solutions without (control) or with 2-mM DTT or 4.3 µg mL^−1^ TM for 2.5 h. Lines: WT Col-0, *aox1a-1*, *ucp1*, *ero1 ero2*. Transcripts: *BiP1/2*, *sbZIP60*, *CNX1*, and *PDI6*. Expression in the mutants relative to WT (raw values in Supplemental Data Set 15); bars above and below black horizontal line indicate expression higher or lower relative to WT, respectively. Mean + sd. *N* = 3 plates with pooled roots from 18 to 20 seedlings each, except for DTT-treated WT and *aox1a-1* (*N* = 2 plates). Differences were tested by two-way ANOVA with Dunnett’s multiple comparisons test (^ns^*P* > 0.05, *P < 0.05, **P < 0.01, ***P < 0.001). B, Primary root length increase of WT and transgenic seedlings (*ero1*, *aox1a*, and *ero1 aox1a-1*). Seedlings were grown vertically on half-strength MS agar plates for 4 days, then transferred to fresh plates supplemented with 0–2,000 µM DTT. Representative images of seedlings 4 days after transfer. Scale bars: 10 mm. C, Root length increase measured 4 days after transfer. 0 µM: *N* = 14–15, 400 µM: *N* = 15, 800 µM: *N* = 15, 2,000 µM: *N* = 15. Boxplot: first and third quartiles with median and Tukey’s whiskers. Differences were tested after log-transformation of data to establish normal distribution. Different letters indicate significant differences (one-way ANOVA with Bonferroni’s multiple comparisons test; *P* < 0.01). *P*-values: Supplemental Data Set 4. D, Rationale of how excess thiol reductant in the form of externally supplied DTT may be detoxified by both the ER through ERO activity and by the mitochondria through respiration.

We next asked whether the root growth phenotypes of the *aox1a* and *ucp1* mutants are causally linked to ER stress responses or are rather independent and due to other effects of reductive stress on cell physiology. In the former case, more severe ER stress is expected in *ucp1* and *aox1a* than in the WT at DTT exposure; in the latter case, severity of ER stress is expected to be similar between genotypes. First, we used acute induction of ER stress as a model, assessing transcript responses of seedling roots after 2.5 h of exposure to 2 mM DTT. *aox1a* and *ucp1* both showed consistently potentiated induction of the ER stress marker transcripts *BiP1/2*, *sbZIP60*, *CNX1*, and *PDI6* to DTT as compared to WT (Figure 4A). The boost in induction was comparable to that in *ero1 ero2*. Redox-independent inhibition of protein maturation by acute exposure to TM (D’Amico et al., 1992; Koizumi et al., 1999) did not lead to an augmented ER stress transcript marker response in *ucp1* and *aox1a*, which was also reflected in similar root growth of all genotypes in the presence of TM (Supplemental Figure S4, G and H). Those data suggest that the DTT-induced phenotype of the mitochondrial mutants is caused by more severe reductive ER stress, indicating that mitochondrial respiration is involved in safeguarding the ER from reductive stress.

We next sought independent validation of the link between mitochondrial respiration and ER stress. We crossed the *ero1-3* background into the *aox1a-1* background and selected homozygous offspring following the rationale that the *ero1* line has decreased thiol oxidation capacity in the ER, as apparent from the characteristic root growth phenotype under DTT challenge (Supplemental Figure S1). This phenotype was synergistically exacerbated in *ero1 aox1a* seedlings as compared to seedlings of the *ero1* and *aox1a* single backgrounds (Figure 4, B and C; Supplemental Figure S5). Those data strongly suggest that AOX1a affects the severity of DTT-mediated reductive stress in the ER lumen (Figure 4D).

### Thiol-based reductive stress leads to NAD reduction in vivo

**Figure 5:**
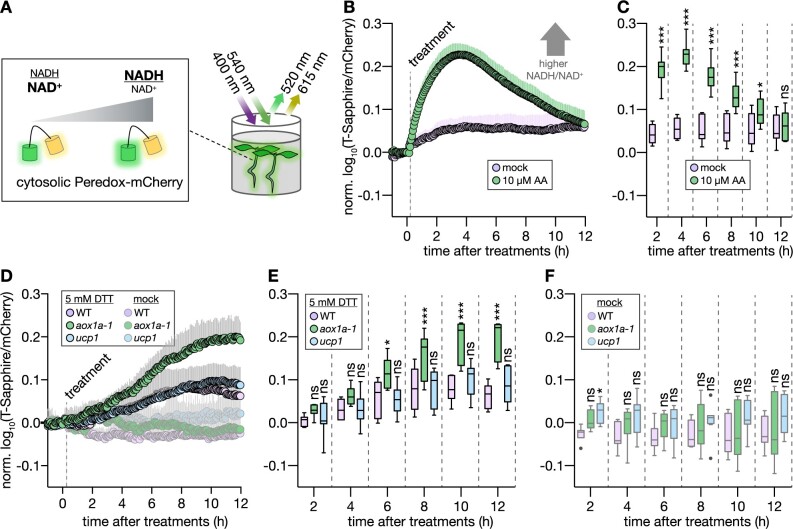
In vivo monitoring of the cytosolic NAD redox state reveals increased NAD reduction in Arabidopsis *aox1a* seedlings following DTT exposure. A, Schematic representation of plate reader-based fluorimetry to monitor NAD redox dynamics in the cytosol of Arabidopsis seedlings. B, Time series of four-day-old WT Col-0 seedlings expressing cytosolic Peredox-mCherry. T-Sapphire and mCherry fluorescence intensities were recorded from seedlings in assay medium and the autofluorescence from corresponding WT controls without sensor were used for background subtraction. T-Sapphire: excited at 400 ± 5 nm, emission collected at 520 ± 5 nm; mCherry: excited at 540 ± 10 nm, emission collected at 615 ± 9 nm. Dashed line indicates addition of mock or AA. High T-Sapphire/mCherry emission ratios indicate a more reduced NAD pool. *N* *=* 7–15. Mean + sd. C, Indicated time points from (B). *N* *=* 7–15. D, Time series as in (B), but with WT and mutants, *aox1a-1* and *ucp1* treated with mock or DTT. E, Indicated time points from (D). *N* *=* 7. F, Indicated time points from (D). *N* *=* 6–8. Boxplots: first and third quartiles with median and Tukey’s whiskers. Significant differences compared to mock treatment (C) or WT (E, F) according to two-way ANOVA with Dunnett’s multiple comparisons test (^ns^*P* > 0.05, *P < 0.05, **P < 0.01, and ***P < 0.001). *P*-values: Supplemental Data Set 5.

A link between mitochondrial respiration and tolerance to reductive ER stress raises the question of how crosstalk between the redox systems of respiratory metabolism and Cys-based redox regulation may occur. While several candidate mechanisms exist to explain how thiol-based reductant may be utilized by the mETC from a thermodynamic and biochemical perspective, it is unclear what mechanism(s) operate at quantitatively meaningful capacity in the living cell. We reasoned that if low-molecular thiols can donate electrons that can reach the mETC, DTT addition may overwhelm baseline respiratory capacity and lead to insufficient oxidation rates of respiratory substrates, such as reduced nicotinamide adenine dinucleotide (NADH). Since the NAD pools of the matrix and the cytosol are major electron donors to the mETC (via the different NAD(P)H dehydrogenases of the inner mitochondrial membrane as well as metabolite shuttle systems), we focused on the cytosolic NAD redox status using a genetically encoded biosensor (Steinbeck et al., 2020). We used acute DTT treatment of living seedlings to make a potential NAD involvement detectable by NADH accumulation (Figure 5A) and we observed pronounced NAD reduction (Figure 5, D–F; Supplemental Figure S6). The acute response to 5 mM DTT was reminiscent of the effect of AA (Figure 5, B and C) and other situations under which mETC capacity becomes limiting relative to respiratory substrate, such as under hypoxia (Wagner et al., 2019; Steinbeck et al., 2020). Interestingly, the absence of *aox1a* exacerbated the sensor response, indicating even stronger NAD reduction and suggestive of limiting mETC capacity to oxidize NADH (Figure 5, D–F). This effect was not detectable in the *ucp1* background, however. Even though the exact mechanism(s) of NAD reduction are unlikely to be straightforward to pinpoint in vivo, the data demonstrate crosstalk between thiol redox systems and metabolic redox systems at rates that have a profound effect on redox physiology.

### mETC activity is directly influenced by electrons derived from DTT

**Figure 6:**
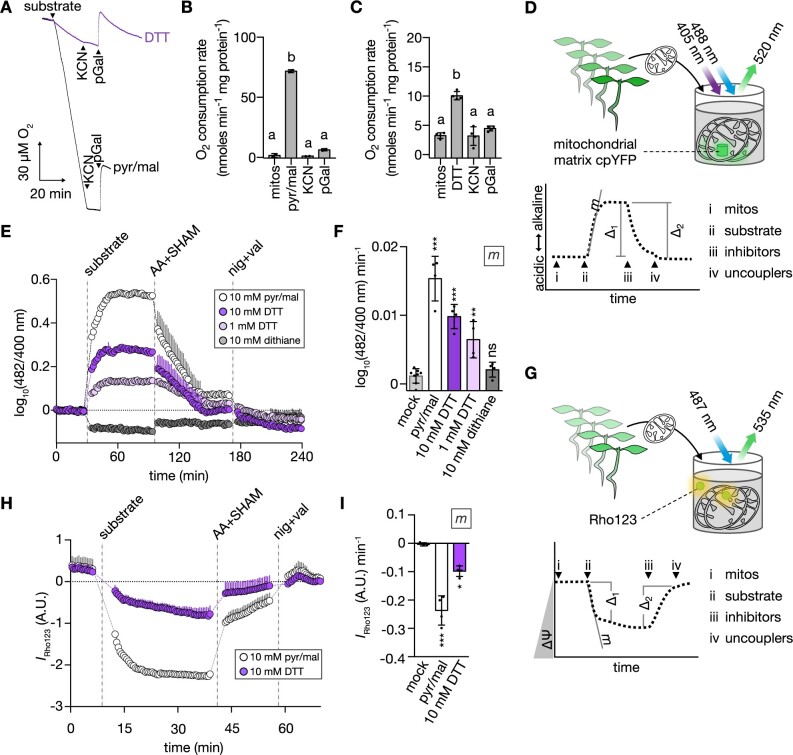
Electrons from thiols can induce oxygen consumption, ΔpH, and ΔΨ in isolated mitochondria. A, Representative polarographic traces from oxygen consumption assays with purified mitochondria from 14-day-old WT Col-0 Arabidopsis seedlings. Arrow heads indicate additions of reagents to mitochondria. Black trace: 10 mM pyruvate + 10 mM malate + 0.3 mM NAD + 0.1 mM thiamine pyrophosphate (substrate; pyr/mal); violet trace: 10 mM DTT (substrate); both traces: 1 mM KCN, 0.2 mM pGal. B and C, Corresponding oxygen consumption rates from (A). Mean ± sd. (B) *N* = 2, (C) *N* = 4. Different letters indicate statistical differences according to repeated measures ANOVA with Tukey’s multiple comparisons test (*P* < 0.01). D, Schematic representation of plate reader-based fluorimetry to monitor pH dynamics in the mitochondrial matrix. (Left) purified mitochondria from 14-day-old Arabidopsis seedlings expressing the mitochondrial matrix-localized cpYFP. CpYFP was sequentially excited at 400 ± 5 nm and 482 ± 8 nm, emission recorded at 520 ± 5 nm. Right: pH dynamics in the mitochondrial matrix in response to i–iv. i: inactive mitochondria. ii: substrates to restore mETC activity and ΔpH across the inner mitochondrial membrane. iii: inhibitors to arrest mETC activity and passively dissipate ΔpH. iv: uncouplers to fully degrade ΔpH. *m*: slope of linear phase in response to substrate addition; Δ_1_: substrate-induced cpYFP ratio difference; Δ_2_: mETC inhibitor-induced cpYFP ratio difference. Δ_1_ and Δ_2_: Supplemental Figure S8, D and E. E, pH dynamics in the mitochondrial matrix as in (D). CpYFP fluorescence ratio depicted as deviation from mean of mock treatment (dotted line at *y* = 0). Increase or decrease of ratio indicate more alkaline or more acidic pH, respectively. *N* = 4. Mean + sd. Vertical dashed lines indicate additions of substrates, mETC inhibitors (AA: 20 μM, SHAM: 2 mM salicylhydroxamic acid) and uncouplers (nig: 50 μM nigericin, val: 10 μM valinomycin). F, Corresponding *m* (slope) from (E). *N* = 4–8. Mean ± sd. G, Schematic representation of the plate reader-based fluorimetry to monitor dynamics of ΔΨ. Sensor-free mitochondria were incubated with the ΔΨ-sensitive dye Rho123. H, ΔΨ dynamics of mitochondria in response to different substrates recorded as described in (G). Rho123 was excited at 487 ± 7 nm, emission collected at 535 ± 15 nm. *N* = 3–4. Mean + sd. Vertical dashed lines as in (E). I, Corresponding *m* (slope) from (H). *N* = 3–4. Mean ± sd. Significant differences between treatments according to one-way ANOVA with Dunnett’s multiple comparisons test (^ns^*P* > 0.05, *P < 0.05, **P < 0.01, ***P < 0.001). *P*-values: Supplemental Data Set 6.

To further examine the role of mitochondria in alleviating thiol-mediated reductive stress, we asked if thiol-derived electrons can be accepted by mitochondrial respiration. Indeed, oxygen consumption analyses using Clark-type electrodes showed DTT-induced oxygen consumption rates indicative of active electron transport. The respiratory activity in response to DTT addition in state II was considerably smaller than for standard respiratory substrates, such as pyruvate/malate. Yet, the respiratory activity was suppressed by potassium cyanide (KCN) and propyl gallate (pGal) inhibiting complex IV and AOX, respectively, arguing against an artifactual rate due to oxidation of DTT by mechanisms independent of the mETC (Figure 6, A–C; Supplemental Figure S7). Considering this surprising finding which on its own needs to be interpreted with caution, we sought independent validation and devised two orthogonal approaches. To monitor pH gradient (ΔpH) dynamics in isolated mitochondria, we made use of the genetically encoded pH sensor cpYFP expressed exclusively in the mitochondrial matrix (Schwarzländer et al., 2011; Figure 6D). The addition of DTT to purified cpYFP-containing mitochondria triggered an immediate alkalinization of the matrix (as indicated by an increase of the cpYFP ratio), indicating proton export by mETC activity (Figure 6E). Inhibiting complex III and AOX, or membrane uncoupling reverted the alkalinization response (Figure 6E; Supplemental Figure S8, D and E). The DTT triggered pH signature was concentration-dependent (1 and 10 mM DTT) and similar to that of pyruvate/malate, albeit at lower amplitudes (Figure 6, E and F; Supplemental Figure S8, D and E). Dithiane failed to elicit a respiratory substrate-like ΔpH response, confirming the thiol group as the causative electron donor. In an analogous set of experiments, we also assessed mitochondrial membrane potential (ΔΨ) using rhodamine 123 (Rho123) quenching (Figure 6G). Consistently, DTT-induced ΔΨ dynamics were similar to those induced by pyruvate/malate but showed a lower amplitude (Figure 6, H and I). Together the data strongly suggest that the mitochondrial respiratory machinery can oxidize and detoxify the DTT thiol.

### Endogenous small thiol molecules can also deliver electrons to the mETC

DTT is a frequently used reducing compound that affects cell physiology via the interaction with endogenous thiols and thereby induces reductive stress. To elucidate whether respiration fuelled by electrons derived from the DTT thiol is a more general property of other low molecular thiols, reduced glutathione (GSH), cysteine (Cys), *N*-acetyl-cysteine (NAC), and β-mercaptoethanol (β-ME) were tested for their ability to generate a proton gradient in isolated mitochondria. Indeed, we observed similar ΔpH dynamics to those induced by pyruvate/malate and DTT, albeit with different amplitudes depending on the specific thiol compound (Supplemental Figure S8, A–E). Only β-ME failed to induce matrix alkalinization. Strikingly, the addition of GSH, which is the main low molecular weight thiol compound in the cytosol, the mitochondria, and the ER and present in millimolar concentrations (Meyer et al., 2001; Zechmann et al., 2008; Krueger et al., 2009; Queval et al., 2011), elicited similar dynamics to DTT (Supplemental Figure S8, A and C–E). Glutathione disulfide (GSSG) failed to induce a respiratory substrate-like response (Supplemental Figure S8, A and C–E). The qualitative effect of the thiol treatments on mitochondrial energization status as well as inhibition by respiratory poisons was strictly reproducible across independent mitochondrial preparations (Supplemental Figure S9). The ability of mitochondrial electron transport to mediate the oxidation of thiols is not unprecedented, considering that disulfide formation for oxidative protein folding in the intermembrane space donates electrons into the mETC (Bihlmaier et al., 2007; Peleh et al., 2017). However, our data suggest relatively high, metabolically relevant rates pointing to considerable thiol detoxification capacity by mitochondria. That argues for a mechanism that involves the reduction of standard mETC substrates as intermediates, such as NAD, prior to electron entry into the mETC.

### Reductive stress tolerance requires functional ANAC017 signaling

**Figure 7:**
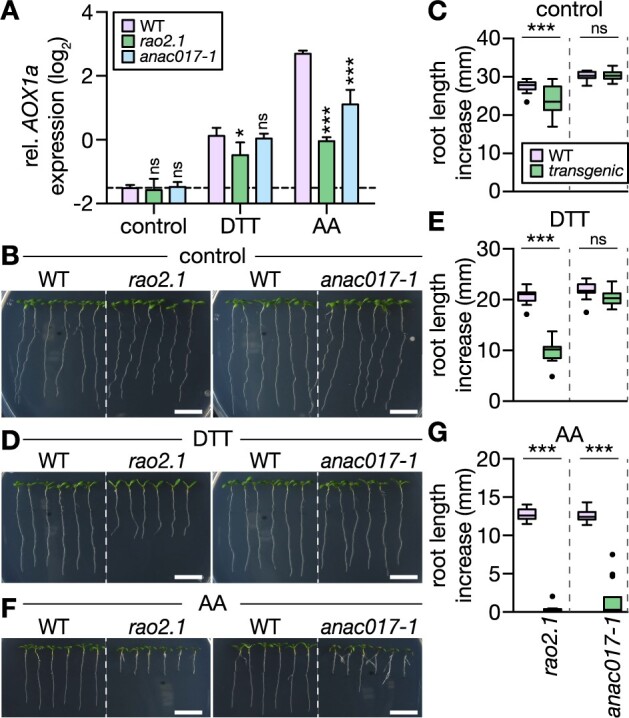
ANAC017-dependent signaling is triggered by DTT and is required to mediate DTT tolerance of root growth. A, *AOX1a* transcript quantification by RT-qPCR from roots of 12-day-old Arabidopsis seedlings submerged in treatment solutions without (control) or with 2 mM DTT or 50 μM AA for 2 h. Lines: WT Col-0, *rao2.1*, *anac017-1*. *AOX1a* expression level relative to reference transcript *TIP41*. Mean + sd. *N* = 3 plates, with pooled roots from 18 to 20 seedlings each. Significant differences to WT according to one-way ANOVA with Dunnett’s multiple comparisons test (^ns^*P* > 0.05, *P < 0.01, and ***P < 0.001). B, D, and F, Primary root length increase of WT, *rao2.1* and *anac017-1* seedlings. Seedlings were grown vertically on half-strength MS agar plates for 4 days, then transferred to fresh plates supplemented with the treatment chemicals. (B) Control; (D) 400 µM DTT; (F) 10 µM AA. Representative images of seedlings 4 days after transfer. Scale bars: 10 mm. C, E, and G, Primary root growth within 4 days after transfer on new plates. (C) *N* = 17–18, (E) *N* = 18, (G) *N* = 17–18. Boxplot: first and third quartiles with median and Tukey’s whiskers. Differences were tested after log-transformation of data to establish normal distribution by one-way ANOVA with Bonferroni’s multiple comparisons test (^ns^*P* > 0.05, ***P < 0.001). *P*-values: Supplemental Data Set 7.

**Figure 8:**
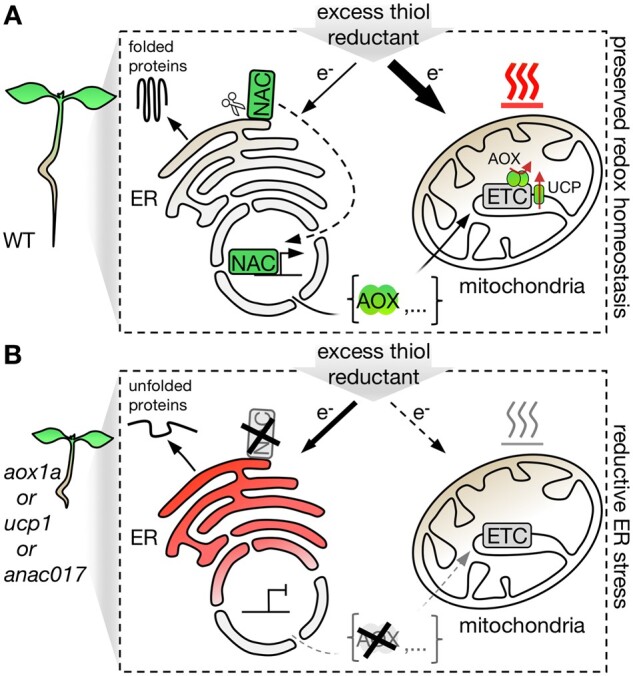
Hypothetical model of how retrograde signaling may mediate redox communication between endoplasmic reticulum and mitochondria to alleviate reductive stress. A, Thiol-based reductant excess is actively dissipated in mitochondria by flexible uncoupling of respiration from oxidative phosphorylation in WT Arabidopsis plants. The respiratory uncoupling capacity is flexibly upregulated via transcriptional regulation of UCP1 and AOX1a, and via ANAC017-dependent retrograde signaling-controlled *AOX1a* expression. B, Decreased mitochondrial uncoupling capacity, for example, lack of functional UCP1 or AOX1a, disables efficient dissipation of excess reductant, leading to reducing ER stress. Similarly, perturbation of the retrograde signaling pathway, for example, lack of functional ANAC017, abolishes the efficient upregulation *AOX1a* expression, thus lowering the dissipation capacity of excess reductant.

DTT, but not TM, treatments of Arabidopsis leaves were previously observed to induce *AOX1a* transcript levels (Supplemental Figure S10, A and B; Martínez and Chrispeels, 2003). The increased *AOX1a* transcription upon DTT treatment was reproducible in roots (Figure 7A). Efficient induction of *AOX1a* expression is regulated via the ER-localized ANAC017 (Ng et al., 2013a). We found that *AOX1a* transcript induction in response to reductive stress was partially suppressed in seedlings of the *ANAC017* mutant *regulators of alternative oxidase1a 2* (*rao2.1*) but not in the independent mutant line *anac017-1* (Figure 7A). A stronger suppression of *AOX1a* expression in *rao2.1* than in *anac017-1* was also observed in the AA treatments (Figure 7A), which is consistent with earlier reports (Ng et al., 2013a). We next examined whether suppressed *AOX1a* induction relates to altered reductive ER stress tolerance. Root length increases of WT, *rao2.1*, and *anac017-1* seedlings were compared in the presence of DTT. Root elongation of *rao2.1* was suppressed by 53% relative to WT (Figure 7, D and E). Only at elevated DTT concentrations, *anac017-1* root elongation was suppressed by 19% relative to WT (Supplemental Figure S11, A and B). Transfer to AA plates strongly suppressed root length increase of both *rao2.1* and *anac017-1* compared to WT. Correlation between root length increase and suppression of *AOX1a* expression (Figure 7, A, D, and E) suggests that ANAC017 signaling is triggered by thiol-mediated reductive stress and is required to alleviate the stress by the induction of *AOX1a* expression (Figure 8).

## Discussion

### A novel phenotype of impaired plant respiratory flexibility

The root growth impairment in *aox1a* plants under electron supply from thiols—as achieved through DTT exposure—links AOX1a to a phenotype that is remarkably pronounced but was missed in previous investigations. *aox1a* mutants were found to display symptoms of elevated stress in green tissues (e.g. the increased accumulation of anthocyanins) under conditions which cause an excess of metabolic reducing equivalents, for example, drought and increased light intensities (Giraud et al., 2008; Kühn et al., 2015; Dahal and Vanlerberghe, 2017). Specific effects of the lack of AOX1a on photosynthetic efficiency have been investigated at mechanistic depth (Bartoli et al., 2005; Dahal et al., 2015; Dahal and Vanlerberghe, 2017, 2018), while observations of AOX1a-related phenotypes under biotic or salt stress have been more loosely linked to the modulation of mitochondrial ROS production (Smith et al., 2009; Vishwakarma et al., 2015; Dahal and Vanlerberghe, 2017). Even though the AOX mode of function supports the idea of a platform role in alleviating mETC flux impairment and maintaining cellular redox balance across a broad range of developmental and environmental fluctuations (Van Aken et al., 2009; Selinski et al., 2018; Florez-Sarasa et al., 2020; Vanlerberghe et al., 2020; Wang et al., 2020), the phenotypes associated with the genetic impairment of AOX function in vivo have been remarkably mild, and limited to specific growth regimes (Giraud et al., 2008; Strodtkötter et al., 2009; Kühn et al., 2015; Oh et al., 2021). Consistent with these mild and specific effects, a range of treatments that we used to induce acute stress at the seedling level did not lead to any obvious phenotypic difference (Supplemental Figure S2). This robustness may be either accounted for by functional redundancy delivered by other AOX isoforms, or by additional cellular mechanisms with similar physiological effect. Simultaneous lack of AOX1a and blockage of classical electron transport, for example, by pharmacological inhibition with AA, however, cannot be tolerated, as evident from root growth arrest in our experimental system (Figure 1C) and independent findings (Strodtkötter et al., 2009; Vishwakarma et al., 2015). Hence, capacity from other AOX isoforms is usually insufficient for compensation in Arabidopsis seedlings. Interestingly, a recent study found that AOX1d can compensate for absence of AOX1a under specific metabolic conditions such as high rates of proline catabolism, as physiologically occurring during recovery from salinity stress (Oh et al., 2021). In *aox1a* seedlings, DTT treatment led to a similar phenotype of impaired root growth as for AA, suggesting mETC capacity became limiting for the dissipation of thiol-mediated reductive stress in the absence of AOX1a. The observation that no other AOX isoform could compensate for the lack of AOX1a was mirrored in the abundance of AOX proteins. No residual signal from other AOX isoform could be detected by Western blotting in *aox1a* roots using an AOX1/2 antibody, suggesting that AOX1a was the dominant AOX isoform in the experimental system by far (Supplemental Figure S10, D and E). The DTT effect in the absence of AOX1a is distinct from the stimulating effects that DTT has on AOX capacity in isolated mitochondria. There DTT is used to reduce an intermolecular disulfide bridge that covalently links AOX dimers. Yet, DTT exposure of seedling roots did not affect the in vivo redox status of AOX1a of WT seedling roots in our experimental system (Supplemental Figure S10, D and E). Instead, AOX1a was maintained in a fully reduced state, which is consistent with previous in vivo observations (Millenaar and Lambers, 2003; Del-Saz et al., 2018; Meyer et al., 2021).

### A shared role for AOX1a and UCP1 in reductive stress alleviation?

UCPs operate downstream of the mETC, but can have a marked effect on its activity, by dissipating the proton motif force across the inner mitochondrial membrane that can limit mETC activity. The similarity of the root growth phenotype between *aox1a* and *ucp1* seedlings in response to DTT (Figure 3) provides correlative evidence for a degree of physiological redundancy between AOX1a and UCP1, even though their biochemical mechanisms are not related, and their regulation differs. Both AOX and UCP proteins can enhance the capacity of mitochondrial respiration to maintain cellular redox homeostasis and avoid metabolic over-reduction (Barreto et al., 2020; Vanlerberghe et al., 2020; Alber and Vanlerberghe, 2021). Indications for in planta uncoupling function of UCP1 have been provided by impaired photorespiration in an Arabidopsis *ucp1* mutant and enhanced drought and salt stress tolerance in *Nicotiana tabacum* UCP1 over-expressors (Sweetlove et al., 2006; Begcy et al., 2011; Barreto et al., 2014, 2017). The uncoupling activity of UCP was recently challenged based on in vitro transport studies after reconstitution of *At*UCP1 into phospholipid vesicles (Monné et al., 2018). The in vitro nature of the analysis, the lack of a membrane potential and absence of biochemical activators that are critical for uncoupling activity, however, makes for an inconclusive case to exclude an uncoupling function, considering the evidence from less reductionist systems, such as functional mitochondria (Smith et al., 2004). While the question of the exact mechanism and significance of UCP function in plants is actively discussed (Barreto et al., 2020), the similarity of the root growth responses of *aox1a* and *ucp1* seedlings to DTT provides a piece of correlative in planta evidence for an uncoupling activity of UCP1 in vivo. During revision of this work, root growth impairment of the *ucp1* line was reported even under control conditions (Arcuri et al., 2021). This phenotype did not occur in the absence of DTT in any of the experiments performed here. It is tempting to speculate, however, that the specific cultivation under which root growth impairment was observed may have induced a reductive challenge from an endogenous source.

### The ER as primary cellular target of thiol-mediated reductive stress

DTT treatment has been widely used to study ER stress (Martínez and Chrispeels, 2003; Iwata et al., 2008; Chen and Brandizzi, 2012; Deng et al., 2013). Although DTT readily passes biological membranes and is not specific for any subcellular localization, its effect on compartments like the cytosol, the peroxisomes, or the mitochondria is limited. There, a large majority of protein Cys and small molecular thiol compounds such as GSH are already highly reduced. In contrast, the secretory pathway entertains a dedicated machinery to induce and maintain Cys oxidation for oxidative protein folding. Newly synthesized proteins in the ER are particularly prone to misfolding when the oxidation capacity is insufficient; already mature proteins with disulfide bridges tend to be more stable (Braakman and Bulleid, 2011; Feige et al., 2018). Few proteins of plasmatic cell compartments also form stable disulfides. For instance, players of Cys-based redox machineries, such as peroxiredoxins, can be maintained in a partially oxidized state since they act as important electron sinks for active Cys-based redox regulation (Vaseghi et al., 2018; Yoshida et al., 2018). Here, DTT is expected to cause reduction, increasing the antioxidant capacity but also interfering with redox regulation. A limited number of structural disulfides are also generated in the chloroplast thylakoid lumen and the mitochondrial intermembrane space, from where disulfide proteins are inserted into respiratory chain complexes (Carrie et al., 2010; Peleh et al., 2017; Meyer et al., 2019). Consequently, an effect of DTT on mitochondrial disulfides cannot be ruled out in root tissues. Yet, plant mitochondrial respiratory function is robust to DTT exposure and DTT is a standard additive for assays with isolated mitochondria to keep Cys of enzymes like AOX1a in their reduced in vivo state (Schwarzländer and Fuchs, 2019; Supplemental Figure S10, D and E). DTT-induced reductive stress was confirmed to affect the ER preferentially (Figure 2; Supplemental Figures S1 and S3A), UPR_ER_ signaling was enhanced in the *aox1a* and *ucp1* backgrounds (Figure 4A), and *ero1 aox1a* seedlings showed synergistic root growth impairment on DTT relative to the respective single mutants, strongly suggesting that elevated ER stress was causative of the root growth phenotype.

### Physiological significance of DTT-induced reductive stress

Model treatments, such as DTT and AA, have proven useful to decipher cellular signaling and biochemical functions, but their significance for understanding physiological situations deserves critical appraisal. Based on shared effect on cellular physiology and overlapping transcriptome responses, we have recently proposed that AA causes cell physiological rearrangements that also occur under hypoxic stress (Wagner et al., 2018, 2019). Reductive ER stress, for which DTT treatment serves as a model, is thought to occur when sudden boosts in secretion capacity are required, for example, at pathogen exposure, or when hypoxia limits the oxidation capacity of the ER (Oldham et al., 2015).

A circumstantial observation from a previous study links thiol-based reductive stress induced by DTT with metabolic overreduction. DTT-treated Arabidopsis leaves exhibited a transcriptome response that showed striking overlap to that observed for *aox1a* leaves exposed to drought and moderate-light stress (Kolbe et al., 2006; Giraud et al., 2008, 2012). This observation independently pinpoints the functional relationship between the metabolic and thiol-based redox systems, which we demonstrate through DTT-induced NAD reduction (Figure 5) and thiol-driven mitochondrial respiration (Figure 6). It further underlines that DTT provides a model for naturally occurring conditions, such as drought and illumination stress, which induce metabolic overreduction and require AOX1a and UCP1 to maintain redox balance (Sweetlove et al., 2006; Giraud et al., 2008).

### A role for mitochondrial respiration to safeguard the ER from reductive stress

It is intriguing to speculate that naturally occurring challenges that modify redox metabolism and require respiratory flexibility, such as drought and moderate light, also give rise to reductive stress in the ER. AA treatment of roots induced the UPR_ER_ marker *sbZIP60* and *PDI6* (while two others did not change; Supplemental Figure S12), providing preliminary indication that changed mitochondrial respiratory capacity may be sufficient to induce ER stress, even in the absence of an additional reductive burden. Mitochondrial backup for ER redox control may be particularly relevant in plants, due to their sessile lifestyle, and particularly effective due to the flexibility of plant mitochondrial respiration. However, there are indications that the principle may be conserved more widely. A role of mitochondrial respiration in alleviating ER stress has recently been proposed for yeast (*Saccharomyces cerevisiae*) and cultured mammalian cells (PCCL3). Increased mitochondrial respiration constituted a critical component of an adaptive ER stress response to TM and DTT (Knupp et al., 2019). ER stress-induced respiratory rates were mediated by enhanced mitochondrial biogenesis increasing mETC capacity (Hijazi et al., 2020). Since *S.* *cerevisiae* and mammals do not contain AOX, induction of mitochondrial biogenesis may be an analogous means of increasing respiratory capacity. While mitochondrial function directly affects redox metabolism in the cytosol, as well as chloroplasts and peroxisomes through metabolite shuttles, there is currently no conclusive concept of how redox metabolism may affect the ER lumen in plants. However, disulfide formation for protein folding in yeast requires a cytosolic thioredoxin, suggesting that endogenous mechanisms are in place for thiol-based electron passage across the ER membrane (Poet et al., 2017). Mechanisms remain to be discovered, and may include transmembrane proteins that mediate electron transfer or the transport of endogenous thiol compounds (Ponsero et al., 2017). For instance, extreme ER stress and disruption of secretion arises in Arabidopsis *gsh2* mutants with impaired GSH synthesis, as a consequence of hyperaccumulation of the thiol-precursor γ‐glutamylcysteine in the ER lumen (Au et al., 2012). The transport dynamics of GSH across the ER membrane, may provide a plausible interaction mechanism in WT plants, but the mechanisms are currently insufficiently understood to allow a mechanistic working model (Meyer et al., 2019).

### Mechanisms of interaction between respiratory metabolism and thiol redox regulation

The question of how mitochondrial respiration can alleviate reductive stress as induced by excess thiol availability comes down to the question of how the metabolic and respiratory redox systems interact with the Cys-based redox machinery of the cell. DTT can directly reduce the different sulfur-based redox systems efficiently by thiol-disulfide exchange without the need for enzymatic catalysis. Hence, DTT exposure will maintain protein Cys and GSH in a highly reduced state. In the ER of living roots, this is apparent by GSH reduction as monitored by roGFP2 and roGFP2iL (Supplemental Figure S3A; Figure 2, A–C). How electrons from thiols may reach respiratory metabolism is less evident. While reduction of NAD(P) and mETC components is thermodynamically feasible, the different chemistries probably require catalysis to reach meaningful rates in vivo. The observation of DTT-induced reduction of cytosolic NAD (Figure 5, D–F) and the finding that thiols can serve as electron source for effective mitochondrial respiration (Figure 6) provide strong evidence that crosstalk between both redox systems can indeed take place in vivo. Which mechanisms dominate in vivo will require future analysis, but there are several endogenous processes that provide compatible biochemistry and conceptual illustration. (1) DTT can reduce ubiquinone in vitro (Kishikawa et al., 2011) and in *Escherichia coli* (Meyrat and von Ballmoos, 2019). While this mechanism offers direct access for thiol-derived electrons into the mETC, to what extent this reaction occurs in vivo is not clear. (2) The MIA40-ERV1 disulfide relay system located in the mitochondrial intermembrane space can oxidize thiols to pass on the electrons to cytochrome *c* of the mETC (Bihlmaier et al., 2007; Peleh et al., 2017). Whether the electron flux that the system can support is sufficient to reach metabolically meaningful rates is unclear, however. (3) Glutahione disulfide reductase or thioredoxin reductase, that link the reduction of Cys-residues and oxidation of NAD(P)H, may be able to operate in reverse when the redox potential of the Cys compound becomes sufficiently reducing (Reichheld et al., 2007; Marty et al., 2009, 2019). Such a situation is physiologically plausible considering the highly reducing states of cytosolic and mitochondrial GSH and thioredoxins in vivo (Meyer et al., 2007; Schwarzländer et al., 2008; Møller et al., 2020). (4) The mechanism of the NAD-dependent 2-ketoacid dehydrogenase complexes of mitochondrial metabolism (including, pyruvate dehydrogenase, α-ketoglutarate dehydrogenase, glycine decarboxylase, and branched-chain ketoacid dehydrogenase) includes a thiol intermediate of the cofactor lipoic acid. Access of small thiol compounds to the bound lipoic acid in the enzyme complex may allow NAD reduction.

The observation that the endogenous thiol compound, GSH, acted as electron source at physiological concentrations in the millimolar range (Supplemental Figures S8 and S9F) raises the possibility that mitochondria can mediate net-oxidation of GSH also in vivo. This would not only provide a physiological mechanism of reductive stress alleviation, but also implicate the mETC in stabilizing the redox status of thiol pools to provide oxidase activity when required, in analogy to the EROs in the ER lumen.

The finding that electrons from thiol oxidation can enter the respiratory chain, possibly by a mechanism that involves reducing NAD(P), expands the role of the mETC as a flexible oxidation platform for the safe disposal of electrons from diverse sources (Gardeström et al., 1980; Planchet et al., 2005; Sweetlove et al., 2006; Chrobok et al., 2016; Welchen et al., 2016; Launay et al., 2019). It further identifies the mitochondrion as an active player in cellular Cys-based redox regulation, where an understanding of the sources of oxidation for reversible Cys-based redox switching has only started to emerge (Yoshida et al., 2019). The extent to which the mETC may act as an electron sink to endogenous thiols, such as GSH, also under nonstress conditions, and the rates at which such a mechanism may operate, deserve further investigation. A steady rate of GSH oxidation by a mechanism that involves the mETC, as balanced by a steady rate of reduction by glutathione disulfide reductases at the expense of metabolically-derived NAD(P)H, may constitute an efficient mechanism to rapidly adjust *E*_GSH_. In such a hypothetical model the mitochondria would make up the downstream section of a pathway that delivers net “GSH oxidase” activity by hosting a steady flux of GSH-derived electrons. Such a concept of GSH redox control does not hinge of the presence of AOX and may be applicable in various eukaryotes also beyond plants.

### A feedback mechanism to alleviate reductive stress by boosting respiratory capacity

The degree of root growth impairment at DTT exposure was similar in the *rao2.1* (EMS mutant impaired in the ANAC017 locus) and the *aox1a* backgrounds. The phenocopy suggests that in WT *AOX1a* expression needs to be induced by ANAC017-mediated signaling to provide protection from reductive stress, and that *AOX1a* is the critical ANAC017 target to confer DTT tolerance. Indeed, DTT was able to induce *AOX1a* transcript abundance and the induction was repressed in the *rao2.1* background (Figure 7). An insertion line affected in the same locus, *anac017-1*, did not show the same repression of both root growth and *AOX1a* transcript induction. This difference between the two lines is consistent with previous observations (Ng et al., 2013a). The quantitative correlation between *AOX1a* transcript induction and root growth further supports that it is the ability to induce *AOX1a* expression that confers reductive stress tolerance. The data give rise to a working model in which an excess of thiol reduction equivalents can induce *AOX1a* expression in an ANAC017-mediated manner (Figure 8). In turn, AOX1a enhances respiratory capacity to accommodate increased electron transport flux to oxygen, while avoiding inhibition through proton motive force build-up or high adenylate charge. As a result, boosted electron transport capacity via AOX as non-proton pumping terminal oxidase supports reductive stress alleviation by oxidizing thiols at rates that are not limited by the energetic status of the cell. If respiratory capacity cannot be sufficiently induced by impaired ANAC017-mediated MRR or insufficient uncoupling in *aox1a* or *ucp1,* the ER is exposed to elevated reductive stress resulting in a stronger UPR_ER_. The regulatory feedback is reminiscent of AA-induced *AOX1a* expression, where complex III inhibition triggers ANAC017 signaling to induce *AOX1a* expression that provides an electron bypass. Most of the currently known players of MRR were identified based on this mechanism (Ng et al., 2013a). Even though our data indicate that *AOX1a* is the most important ANAC017 target with respect to reductive stress alleviation, ANAC017-based MRR regulates an entire set of nuclear genes in addition (De Clercq et al., 2013). Of the 100 genes with the strongest transcript fold increases at AA exposure and positive regulation through ANAC017 function reported by Ng et al. (2013a), 26 were also identified in a transcriptome analysis in response to DTT (Martínez and Chrispeels, 2003) and a majority showed upregulated expression in response to DTT (Supplemental Figure S10). Functional crosstalk between the mitochondria and the ER has further been evidenced by increased *bZIP60* expression in response to AA treatment and in ANAC017 overexpressing lines (Meng et al., 2019); vice versa in the *ire1a ire1b* line increased expression of *AOX1a* was observed (Ng et al., 2013a, 2013b). While the biochemical mechanisms that may underpin this crosstalk remain to be investigated in plants, future progress may be guided by recent examples for the functional interaction between mitochondrial and ER stress programs in non-plant systems (Neal et al., 2017; Knöringer et al., 2021).

### Reductive stress as upstream trigger of MRR?

The overlapping fingerprints of AA and DTT raise the possibility that not only ANAC017 signaling is triggered by both, but that also the upstream stimuli may be shared. Since AA stimulates superoxide generation from complex III of the mETC, that is rapidly converted to H_2_O_2_, mitochondrial ROS have been proposed as a messenger in ANAC017-mediated MRR. As a net electron donor to the mETC, DTT may also stimulate mitochondrial ROS production (Figure 6). However, DTT also acts as a potent electron donor for the thiol-based antioxidant systems (Schwarzländer et al., 2008). Indeed, the induction of mitochondrial ROS production is just one out of several different physiological consequences of AA treatment (Wagner et al., 2019). For instance, NAD reduction is a shared response to AA and DTT (Figure 5), highlighting the possibility that reductive stress, based on NAD-thiol crosstalk, may also play a mechanistic role in triggering MRR. That said, reductive stress and increased mitochondrial ROS production are not mutually exclusive and may rather be regarded as two sides of the same coin (Loscalzo, 2016). Limited mETC flux causes reduction of redox couples upstream (e.g. NAD(P)), as well as of mETC redox centers, leading to increased single electron flux to molecular oxygen to generate superoxide and H_2_O_2_ in turn (Murphy, 2009; Huang et al., 2016). Since ANAC017 is a tail-anchor protein in the ER membrane that is mobilized to the nucleus through cleavage by a yet unknown rhomboid protease, the question of upstream regulation boils down to the regulation of the proteolytic event (Finkemeier and Schwarzländer, 2018). The susceptibility of the ER to reductive stress would provide an attractive mechanism, for instance by reduction of a lumen-exposed disulfide, or NADH binding by a cytosol-exposed binding site. Interestingly, subcellular Ca^2+^ dynamics were previously suggested to connect UPR_ER_ and MRR under salt stress depending on *At*WRKY15 expression (Vanderauwera et al., 2012).

We recently observed that hypoxia stress induces shared subsets of transcripts as AA (Wagner et al., 2018), based on which we proposed that hypoxia stress provides a physiologically relevant situation for the activation of MRR. The physiological signatures of cytosolic MgATP^2-^, NADH/NAD^+^, pH, free Ca^2+^ and *E*_GSH_ were also remarkably similar for AA and hypoxia (Wagner et al., 2019). Independently, ANAC017 signaling was observed to be important for submergence tolerance, which includes hypoxia stress (Meng et al., 2019; Bui et al., 2020). Since hypoxia stress entails a shortage in oxygen as terminal electron acceptor, it represents a situation of electron excess, i.e. a reductive shift of key redox systems such as NADH/NAD^+^. While mETC is particularly affected, also oxidative protein folding in the ER relies on oxygen, as mediated by ERO (Meyer et al., 2019; Ugalde et al., 2021a). Hence hypoxia has been associated with reductive ER stress, similarly to the impact of DTT that was used as a model here.

Taken together, our data strengthen the case that redox signals operate upstream in MRR; those may include ROS-based mechanisms, but the tight biochemical linkage in subcellular metabolism highlights reductive stimuli as likely additional candidates.

## Materials and methods

### Plant materials

The *Arabidopsis thaliana* T-DNA insertion lines *aox1a-1* (SALK_084897; Giraud et al., 2008), *aox1a-2* (SAIL_030_D08; Col-0 background; Giraud et al., 2008), *ucp1-1* (SAIL_536_G01 and the corresponding complemented lines, *cUCP1#9* and *cUCP#14*, Sweetlove et al., 2006), *anac017-1* (SALK_022174; Ng et al., 2013a, 2013b), the EMS mutant *rao2.1* (Ng et al., 2013a, 2013b), *ero1-3* (SALK_096805; Fan et al., 2019; Ugalde et al., 2021a) and the UPR_ER_ double mutants *ire1a ire1b* (SALK_132285, SALK_050203; Chen and Brandizzi, 2012) and *bzip28 bzip60* (WiscDsLox420D09, SAIL_238_F07; Deng et al., 2013) were described previously and validated by genotyping and sequencing before usage. As part of the molecular validation of the *ucp1-1* background we mapped the T-DNA insertion site more upstream than previously reported (Sweetlove et al., 2006), that is, in the promoter region of the *UCP1* locus (Supplemental Figure S13A). Assessment of the transcript levels revealed that the full-length *UCP1* transcript was reduced by at least 80%, but still detectable (Supplemental Figure S13, B–D). Hence, *ucp1-1* represents a knockdown, rather than a knockout line, which is consistent with the immunoblotting analysis for UCP1 protein abundance reported previously (Sweetlove et al., 2006) as well as a recent report (Arcuri et al., 2021). For *ero1 ero2*, the T-DNA insertion line *ero1-3* was transformed to express an amiRNA targeted against *ERO2* (amiRNA hairpin; Ugalde et al., 2021a). A homozygous *ero1 aox1a-1* line was generated by crossing the corresponding homozygous single mutants followed by selection of homozygous individuals in the F2 generation by genotyping. *Arabidopsis thaliana* expressing the cytosolic Peredox-mCherry NADH/NAD^+^ sensor, the ER-localized Grx-roGFP2iL and the mitochondrial matrix cpYFP pH sensor are described in Steinbeck et al. (2020), Ugalde et al. (2021b), and Schwarzländer et al. (2011), respectively. *Ucp1-1* and *aox1a-1* lines expressing the cytosolic Peredox-mCherry sensor were generated by Agrobacterium-mediated transformation via floral dip as described in Steinbeck et al., 2020. Arabidopsis lines expressing roGFP2 biosensors in the ER lumen (CH1-roGFP2-HDEL; Brach et al., 2009), the mitochondrial matrix (SHMT-roGFP2-Grx1; Albrecht et al., 2014), the plastid stroma (TKTP-Grx1-roGFP2; Ugalde et al., 2021b), the peroxisomes (Grx1-roGFP2-SKL; Rosenwasser et al., 2011), and the cytosol (Grx1-roGFP2; Marty et al., 2009) were previously introduced and validated for fluorescence before use.

### Plant phenotyping

Surface-sterilization, stratification of seeds and growth of seedlings under long-day conditions (16 h at 120µmol photons m^−2^ s^−1^ using Osram L18W840 Lumilux cool-white or Philips TL5 HO 49W 840 [MASTER] cool-white tubes at 22°C, 8 h dark at 18°C) on vertical half-strength Murashige and Skoog (MS) medium (Murashige and Skoog, 1962) +0.1% (w/v) sucrose +1.0% (w/v) agar plates (25 mL per plastic dish) was done as detailed in Wagner et al. (2015a, 2015b). For pH stabilization at 5.8 (KOH), culture plate medium was supplemented with 2 mM 2-(N-morpholino)ethanesulfonic acid (Carl Roth, Karlsruhe, Germany). After 4 days growth, seedlings were carefully transferred onto new agar plates supplemented with indicated reagents. Freshly autoclaved medium was cooled to exactly 50°C, supplemented with sterile-filtered reagents and immediately poured into petri dishes for rapid cool down at room temperature. For standardization, DTT stock solutions were prepared, aliquoted, and immediately frozen in liquid nitrogen and stored at −86°C. Aliquots no older than 1 month were thawed on ice before usage. Seedlings mutant lines and the corresponding WT were placed side-by-side on the freshly prepared plates. Plates were sealed with Micropore surgical tape (3M) and returned to long-day conditions. At indicated time points, seedlings were subjected to photographic documentation. Increase of primary root and total root length were quantified from images with Fiji (www.fiji.sc) (Schindelin et al., 2012) and RootReader2D (www.plantmineralnutrition.net/rootreader.htm) (Clark et al., 2013).

### Reverse transcription-quantitative PCR

RNA extracted from seedling roots was reverse-transcribed using the RevertAid First Strand cDNA Synthesis kit (Thermo Fisher Scientific, Waltham, MA, USA) with random hexamer primers. Primer pairs for assessment of transcript abundances in RT-qPCRs were designed according to earlier reports or following the recommendations of Udvardi et al. (2008) (see Supplemental Table S1 for primer sequences). PCR efficiency for each primer pair was assessed by calibration dilution curves in each quantification experiment and quantified as described in Bustin et al. (2009); *10^−1^*^/slope^  *–1*. Primer efficiencies were between 88% and 96%, *R*^2^ > 0.99. Amounts of 1 μL cDNA (25 ng RNA) served as template for RT-qPCRs performed with PerfeCTa SYBR Green FastMix (Quantabio, Beverly, MA, USA) in a 384-well plate using a CFX96 Real-Time PCR Detection System (Bio-Rad, Hercules, CA, USA). All RT-qPCR quantifications were based on three technical replicates and three biological replicates unless stated otherwise. Expression levels were calculated relative to *TAP42 INTERACTING PROTEIN OF 41 KDA* (*TIP41*; *AT4G34270*) (Czechowski et al., 2005), and relative to *ACTIN8* (*Act8*; *AT1G49240*) (Lai et al., 2018) to verify results.

### Mitochondrial isolations

Arabidopsis mitochondria were isolated from 14-day-old Col-0 seedlings as previously described (Escobar et al., 2006). Mitochondrial integrity was verified by outer mitochondrial membrane integrity ≥90% as estimated by cytochrome *c* latency assays (Sweetlove et al., 2007).

### Monitoring of mitochondrial respiration

Oxygen consumption of isolated mitochondria was measured using Oxytherm Clark-type electrodes (Hansatech, King's Lynn, UK) as previously described (Sweetlove et al., 2007; Wagner et al., 2015a). Respiration of 40 μL purified mitochondria (∼4 g L^−1^ mitochondrial protein) was measured in 1 mL basic incubation medium (BIM). mETC inhibitors: 1 mM KCN (Sigma-Aldrich, St Louis, MO, USA), 0.2 mM propylgallate (Sigma-Aldrich, St Louis, MO, USA).

### Multi-well plate reader-based fluorimetry

In organello monitoring of mitochondrial matrix pH dynamics was performed as described previously with slight modifications (Schwarzländer et al., 2011). An aliquot of 10 μL suspension of freshly isolated mitochondria (∼4 g L^−1^ mitochondrial protein) from 14-day-old Arabidopsis seedlings stably expressing mitochondrial matrix-localized circularly permuted Yellow Fluorescent Protein (cpYFP) were added to 190 μL BIM in a transparent 96-well plate (NUNC). Fluorescence of cpYFP was recorded with a CLARIOstar microplate reader (BMG LABTECH, Ortenberg, Germany) at 25°C employing bottom optics with 3.5 mm focal height, well-multichromatic monitoring, 40 flashes per cycle, and double orbital shaking at 500 rpm for 5 s before each measurement cycle. Emission was recorded at 520 ± 5 nm in two separate tracks exciting at 400 ± 5 nm or 482 ± 8 nm. For sequential supplementation, monitoring was briefly paused and appropriate volumes of reagents were manually added. All solutions were prepared in advance, pH-adjusted, and aliquots stored at –86°C. For better comparability of independent experiments, log_10_ ratio value (*I*_482_ _nm_/*I*_400_ _nm_) of mock treatment was subtracted from log_10_ ratio value of treatments. mETC inhibitors: 50 μM AA from *Streptomyces* sp. (Sigma-Aldrich) and 2 mM salicylhydroxamic acid (Sigma-Aldrich). Ionophores: 50 μM nigericin sodium salt (Sigma-Aldrich), 10 μM valinomycin from *Streptomyces fulvissimus* (Abcam, Cambridge, UK). *In organello I*_Rho123_-based monitoring of ΔΨ was optimized based on previous reports (Emaus et al., 1986; Nie et al., 2015). An aliquot of 7.5 μL freshly isolated mitochondria (∼4 g L^−1^ mitochondrial protein) from 14-day-old WT Arabidopsis seedlings were added to 192.5 μL BIM supplemented with 150 nM Rhodamine 123 (ACROS Organics) and added to a transparent 96-well plate (NUNC). The measurements were performed as for cpYFP, except for excitation at 487 ± 14 nm and emission at 535 ± 15 nm. In vivo experiments with four-day-old Arabidopsis seedlings expressing the cytosolic Peredox-mCherry NAD redox sensor were performed by plate reader-based fluorimetry as described in Steinbeck et al. (2020) except that black 96-well plates were used; T-Sapphire: excited at 400 ± 5 nm, emission collected at 520 ± 5 nm; mCherry: excited at 540 ± 10 nm, emission collected at 615 ± 9 nm. In vivo experiments with four-day-old Arabidopsis seedlings expressing the roGFP2 sensors in different subcellular compartments were performed similarly to the report by Wagner et al. (2019); roGFP2 emission was recorded at 520 ± 5 nm in two separate tracks exciting at 400 ± 5 nm or 482 ± 8 nm.

### Confocal laser scanning microscopy

Confocal laser scanning microscopy (CLSM) was performed as previously described (Wagner et al., 2015b) using a Zeiss LSM780 confocal microscope and a ×10 (Plan-Apochromat, 0.3 N.A.) or ×25 lens (LD LCI Plan-Apochromat Imm Korr DIC M27, 0.8 N.A., water immersion). RoGFP2iL was excited at 405 and 488 nm; auto-fluorescence and roGFP2iL fluorescence were collected at 430–470 nm and 508–535 nm, respectively. For time series imaging, Arabidopsis seedlings were mounted in a custom perfusion chamber and continuously perfused (∼1 mL min^−1^) with water, H_2_O_2_, or DTT at indicated concentrations and sequence.

### Ratiometric image data analysis

Ratiometric images and time series data of roGFP2iL recorded at CLSM were analyzed and pseudo-colored using a custom MatLab program package (Fricker, 2016) as described previously for roGFP2 (Attacha et al., 2017).

### Immunoblot analysis

Protein was extracted from roots of 18-day-old Arabidopsis seedlings in 30 mM Tris pH 7.9 (HCl), 1 mM EDTA on ice in the presence of 0.1% plant protease inhibitor cocktail (P9599; Sigma, St Louis, MO, USA), and 20 mM *N*-ethylmaleimide to block all sulfhydryl groups. Protein content was quantified by a Bradford assay (Roti Quant; Carl Roth, Karlsruhe, Germany) and 25 μg protein per sample was separated by nonreducing SDS–PAGE using a 4%–20% gradient gel (4%–20% Mini-PROTEAN TGX Precast Protein Gel; Bio-Rad, Hercules, CA, USA) and either Coomassie-stained in PageBlue Protein Staining Solution (Thermo Scientific, Waltham, MA, USA) or transferred to a polyvinylidene difluoride (PVDF)membrane (Immobilon-P, Millipore Corporation, Billerica, MA, USA) via a semi-dry western blotting system (Trans-Blot SD semi-dry transfer cell; Bio-Rad, Hercules, CA, USA). Membranes were incubated in 5% (w/v) milk powder dissolved in TBS-T (20 mM Tris pH 7.6 (HCl), 137 mM NaCl, 0.1% v/v Tween) overnight at 4°C. Antibodies AOX1/2 (AS04 054, Agrisera, Vännäs, Sweden) and goat anti-rabbit (AS09 602; Agrisera, Vännäs, Sweden) were diluted in TBS-T following the recommendations of the supplier and consecutively used for incubating the membrane for 1 h. Between the incubations and before imaging, the membrane was washed 3 × 5 min each in TBS-T. For detection, the ECL super bright kit (Agrisera, Vännäs, Sweden) was used according to the manufacturer’s instructions. After 2 min of incubation chemiluminescence was detected using a Chemostar ECL imager (INTAS Science Imaging, Göttingen, Germany).

### Statistical analysis

Statistical analyses were performed as described in the individual figure legends using the software Prism version 7.0a (GraphPad Holdings, San Diego, CA, USA; Supplemental Data Sets 1–19).

### Accession numbers

Sequence data from this article can be found in the GenBank data libraries under the following accession numbers: *ACT8*, *AT1G49240*; *AOX1a*, *AT3G22370*; *BiP1*, *ATG28540 BiP2*, *AT5G42020*; *bZIP28*, *AT3G10800*; *bZIP60*, *AT1G42990*; *CNX1*, *AT5G61790*; *ERO1*, *AT1G72280*; *ERO2*, *AT2G38960*; *IRE1a*, *AT2G17520*; *IRE1b*, *AT5G24360*; *ANAC017*, *AT1G34190*; *PDI6*, *AT1G77510*; *TIP41*, *AT4G34270*, *and UCP1*, *AT3G54110*.

## Supplemental data 

The following materials are available in the online version of this article.

**Supplemental Figure S1.** Arabidopsis seedlings show genotype-specific root growth impairments at different DTT concentrations.

**Supplemental Figure S2.** Analysis of root growth increase of Arabidopsis *aox1a* seedlings at exposure to treatment chemicals.

**Supplemental Figure S3.** DTT and dithiane cause reductive ER stress in Arabidopsis seedling roots.

**Supplemental Figure S4.** Arabidopsis *aox1a* and *ucp1* seedlings show genotype-specific root growth impairments at different DTT concentrations.

**Supplemental Figure S5.** Experimental replicate on primary root length increase of WT and transgenic seedlings (*ero1*, *aox1a*, and *ero1 aox1a-1*).

**Supplemental Figure S6.** In vivo monitoring of the cytosolic NAD redox state reveals increased NAD reduction following DTT exposure.

**Supplemental Figure S7.** DTT induces low rates of oxygen consumption in the presence of isolated mitochondria.

**Supplemental Figure S8.** Electrons from small thiol molecules can serve as respiratory substrate to induce ΔpH in isolated mitochondria.

**Supplemental Figure S9.** Independent replicates confirming mETC-dependent induction of ΔpH in isolated mitochondria by small thiol molecules.

**Supplemental Figure S10.** Exposure to DTT causes induction of ANAC017-regulated genes, while AOX1a protein shows no redox changes at DTT treatment in vivo.

**Supplemental Figure S11.** ANAC017-dependent signaling is also required to mediate tolerance of root growth to high DTT concentration.

**Supplemental Figure S12.** mETC inhibition induces ER stress markers.

**Supplemental Figure S13.** Molecular characterization of *ucp1*.

**Supplemental Table S1.** List of primers.

**Supplemental Data Set 1.** Statistical analyses of Figure 1.

**Supplemental Data Set 2.** Statistical analyses of Figure 2.

**Supplemental Data Set 3.** Statistical analyses of Figure 3.

**Supplemental Data Set 4.** Statistical analyses of Figure 4.

**Supplemental Data Set 5.** Statistical analyses of Figure 5.

**Supplemental Data Set 6.** Statistical analyses of Figure 6.

**Supplemental Data Set 7.** Statistical analyses of Figure 7.

**Supplemental Data Set 8.** Statistical analyses of Supplemental Figure S1.

**Supplemental Data Set 9.** Statistical analyses of Supplemental Figure S2.

**Supplemental Data Set 10.** Statistical analyses of Supplemental Figure S3.

**Supplemental Data Set 11.** Statistical analyses of Supplemental Figure S4.

**Supplemental Data Set 12.** Statistical analyses of Supplemental Figure S5.

**Supplemental Data Set 13.** Statistical analyses of Supplemental Figure S6.

**Supplemental Data Set 14.** Statistical analyses of Supplemental Figure S7.

**Supplemental Data Set 15.** Statistical analyses of Supplemental Figure S8.

**Supplemental Data Set 16.** Statistical analyses of Supplemental Figure S11.

**Supplemental Data Set 17.** Statistical analyses of Supplemental Figure S12.

**Supplemental Data Set 18.** Statistical analyses of Supplemental Figure S13.

**Supplemental Data Set 19.** Separated WT and mutant lines ER stress marker transcript quantification values of Figure 4A.

## Supplementary Material

koac017_Supplementary_Data

## Notes

P.F. and M.S. designed the research. P.F., F.B., S.L., J.M.U., E.F.A., and B.M. performed the experiments; P.F., F.B., S.L., C.R., S.W., and M.S. analyzed data; C.R. and A.J.M. provided novel resources, S.W., S.J.M.S, A.J.M., and M.S. supervised the research; P.F. and M.S. wrote the paper and generated the figures. M.S. agrees to serve as the author responsible for contact and ensures communication.

The author responsible for distribution of materials integral to the findings presented in this article in accordance with the policy described in the Instructions for Authors (https://academic.oup.com/plcell) is: Markus Schwarzländer (markus.schwarzlander@uni-muenster.de).
