# Correction to: Reductive stress triggers ANAC017-mediated retrograde signaling to safeguard the endoplasmic reticulum by boosting mitochondrial respiratory capacity

**DOI:** 10.1093/plcell/koac090

**Published:** 2022-04-01

**Authors:** Philippe Fuchs, Finja Bohle, Sophie Lichtenauer, José Manuel Ugalde, Elias Feitosa Araujo, Berivan Mansuroglu, Cristina Ruberti, Stephan Wagner, Stefanie J Müller-Schüssele, Andreas J Meyer, Markus Schwarzländer

*The Plant Cell*, koac017, https://doi.org/10.1093/plcell/koac017

The originally published version of this article mistakenly omitted some proof edits requested by the authors to the supplemental material and omitted two Supplemental files. This has now been corrected upon request of the authors.

In the originally published version of this manuscript, there was an error in the legend of Supplemental Figure S4, where an Arabidopsis mutant line was referred to as “ero1-2.” The correct name is “ero1-3.” This has been corrected.

In addition, due to severe compression applied by the journal, figures in the Supplemental Data accompanying the original published version were difficult to interpret, and the loss of resolution introduced additional artifacts. A higher-resolution file has been added to the supplementary data in its place.

Finally, two files that were supposed to be published with supplemental material were omitted from the published version: the Author Revisions Checklist and the Peer Review Report. These have now been included.

