# Supplementary material for: Reductive stress triggers ANAC017-mediated retrograde signaling to safeguard the endoplasmic reticulum by boosting mitochondrial respiratory capacity
Source: Plant Cell. 2022 Jan 25;34(4):1375–95. doi: 10.1093/plcell/koac017 (PMC9125394; doi:10.1093/plcell/koac017)
Supplement: koac017_Supplementary_Data [file koac017_supplementary_data.zip › 220203_Supplemental figures_Fuchs.pdf]

Research Article

Reductive stress triggers ANAC017-mediated retrograde signaling to safeguard the endoplasmic reticulum by boosting mitochondrial respiratory capacity

SUPPLEMENTAL DATA

Supplemental Information includes 13 Figures, 1 Table and 19 Data Sets, and can be found with this article online.

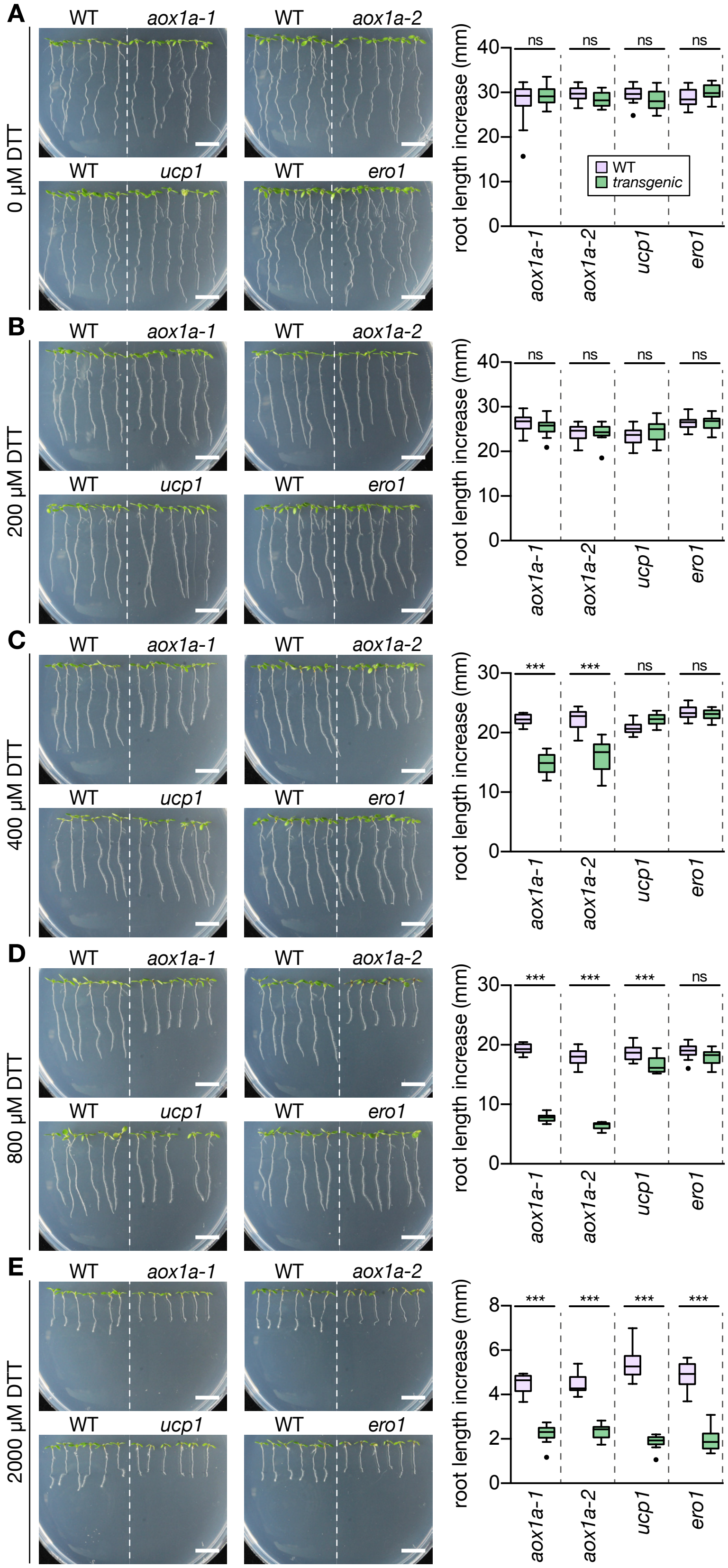

A
B
C
D
E
WT
aox1a-1
aox1a-2
ucp1
ero1
0 µM DTT
200 µM DTT
400 µM DTT
800 µM DTT
2000 µM DTT
root length increase (mm)
ns
***
WT
transgenic

Supplemental Figure S1. Arabidopsis seedlings show genotype-specific root growth impairments at different DTT concentrations (Supports Figures 1, 3 and 4).

Primary root length increase of wild-type Col-0 (WT) and transgenic seedlings (*aox1a*: *alternative oxidase 1a*, *ero1*: *ER oxidoreductin 1*, *ucp1*: *uncoupling protein 1*). Seedlings were grown vertically on half-strength MS agar plates for 4 d, then transferred to fresh plates supplemented with 0–2000 µM dithiothreitol (DTT). Left: Representative images of seedlings 4 d after transfer. Scale bars: 10 mm. Right: Root length increase measured 4 d after transfer. (**A**) 0 µM: *N* = 18. (**B**) 200 µM: *N* = 18. (**C**) 400 µM: *N* = 17–18. (**D**) 800 µM: *N* = 17–18. (**E**) 2000 µM: *N* = 17–18. Boxplot: 1st and 3rd quartiles with median and Tukey whiskers. Differences were tested after log-transformation of data to establish normal distribution by one-way ANOVA with Bonferroni's multiple comparisons test ($^{ns}P > 0.05$, $^{*}P < 0.05$, $^{***}P < 0.001$). *P*-values: Supplemental Data Set **S8**.

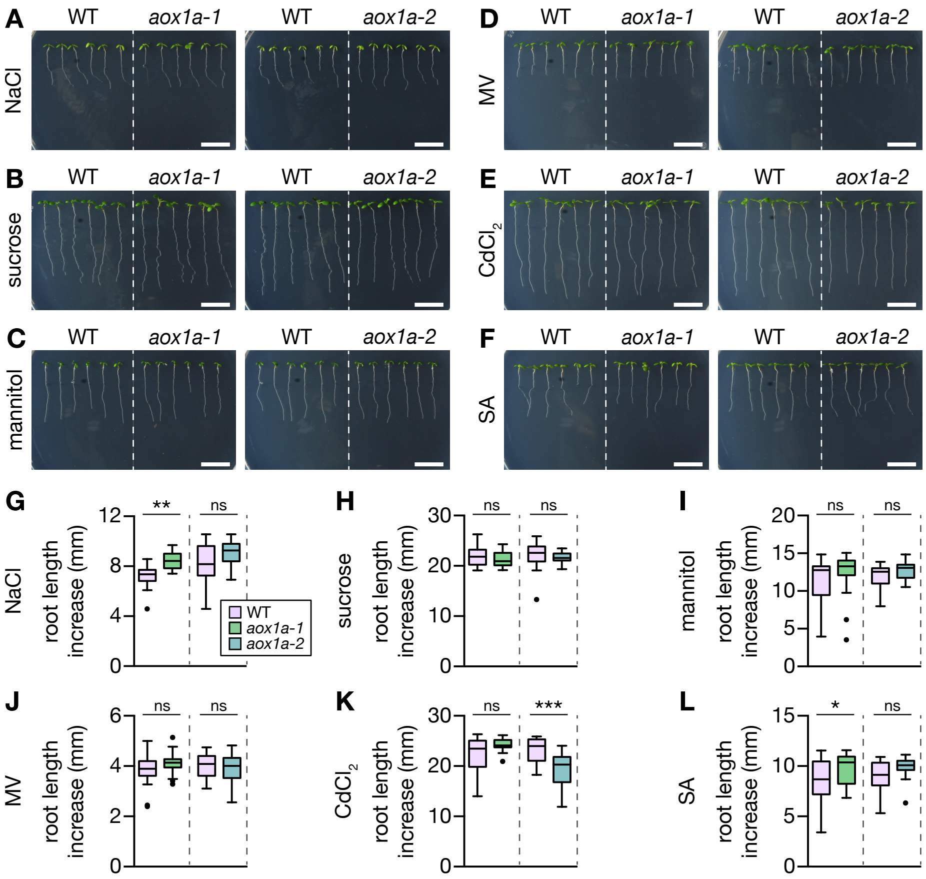

Supplemental Figure S2. Analysis of root growth increase of Arabidopsis *aox1a* seedlings at exposure to treatment chemicals (Supports Figure 1).

Primary root length increase of wild-type Col-0 (WT) and *alternative oxidase 1a* mutant (*aox1a-1* and *aox1a-2*) seedlings. Seedlings were grown vertically on half-strength MS agar plates for 4 d, then transferred to fresh plates supplemented with the treatment chemicals. (**A**) 100 mM NaCl: sodium chloride, (**B**) 5% sucrose, (**C**) 200 mM mannitol, (**D**) 0.1 µM MV: methyl viologen, (**E**) 5 µM $CdCl_2$: cadmium chloride or (**F**) 100 µM SA: salicylic acid. Representative images of seedlings 4 d after transfer to new plates. Corresponding control treatment shown in Figure 1A. Scale bars: 10 mm. (**G**–**L**) Primary root growth within 4 d after transfer on new plates. (G) N = 15–18, (H) N = 18, (I) N = 16–18, (J) N = 18, (K) N = 16–18, (L) N = 12–18. Boxplot: 1st and 3rd quartiles with median and Tukey whiskers. Differences were tested after log-transformation of data to establish normal distribution by one-way ANOVA with Bonferroni's multiple comparisons test ($^{ns}P > 0.05$, $^{*}P < 0.05$, $^{**}P < 0.01$, $^{***}P < 0.001$). P-values: Supplemental Data Set **S9**.

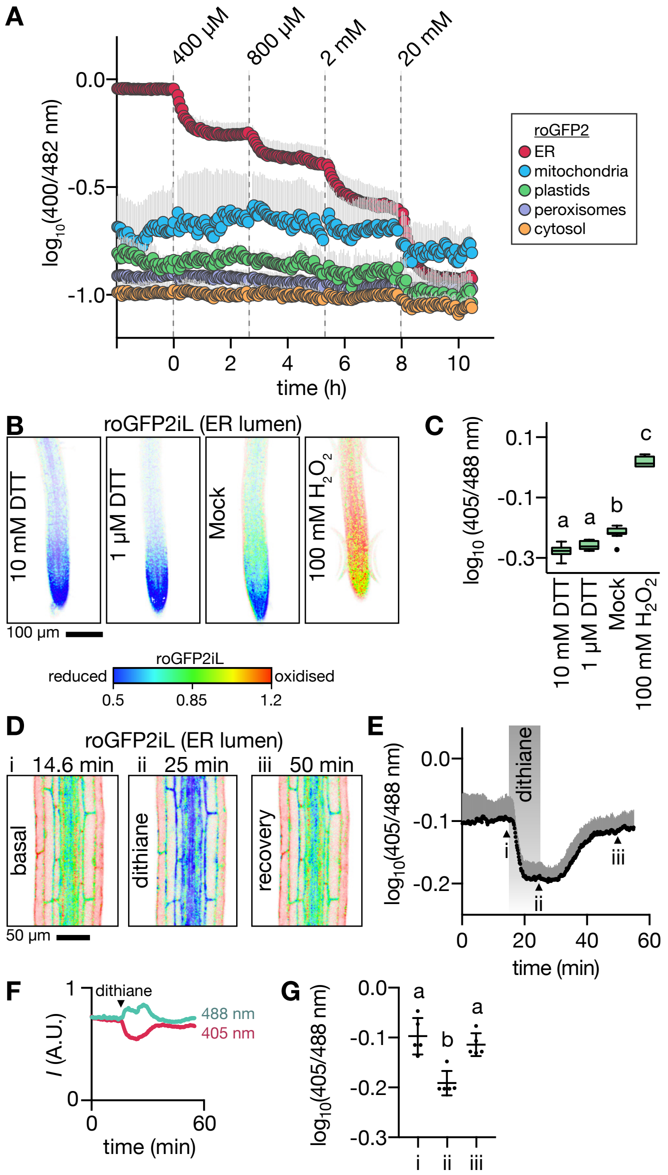

Supplemental Figure S3. DTT and dithiane cause reductive ER stress in Arabidopsis seedling roots (Supports Figure 2).

(**A**) Time series of 4-d-old wild-type Col-0 (WT) seedlings expressing roGFP2 targeted to different cellular subcompartments. roGFP2 fluorescence intensities were recorded from seedlings in assay medium in a plate reader and the autofluorescence from corresponding WT controls without sensor were used for subtraction. Emission was recorded at 530 ± 5 nm in two separate tracks exciting at 400 ± 5nm or 482 ± 8 nm. Dashed lines indicate addition of dithiothreitol (DTT) to the indicated total concentrations in the assay medium. High 400/482 nm excitation ratios indicate a less negative E_{GSH}. N = 7–8. ER: CH1-roGFP2-HDEL, mitochondria: SHMT-roGFP2-Grx1, plastids: TKTP-Grx1-roGFP2, peroxisomes: Grx1-roGFP2-SKL, cytosol: Grx1-roGFP2. (**B**) Representative images of 5-d-old Arabidopsis seedling root tips expressing ER lumen-targeted Grx1-roGFP2iL. Seedlings were incubated in H_2O without (control) or with supplements (DTT, hydrogen peroxide: H_2O_2) at indicated concentrations for 5 min. Images were recorded at the CLSM, ratiometrically analysed and pseudo-coloured. High 405/488 nm excitation ratios indicate a less negative E_{GSH} (red), low ratios indicate a more negative E_{GSH} (blue). (**C**) Corresponding roGFP2iL log_{10} ratio values. Boxplot: 1st and 3rd quartiles with median and Tukey whiskers. N = 6–11. Different letters indicate statistical

differences according to one-way ANOVA with Tukey's multiple comparisons test (P < 0.01). (**D**) Representative time course images of 4-d-old Arabidopsis seedling root expressing ER lumen-targeted Grx1-roGFP2iL. After 15 min, seedlings were perfused with 5 mM trans-4,5-dihydroxy-1,2-dithiane (dithiane) for 5 min. Images were recorded and processed as in (A). (**E**) Corresponding time series. N = 5. Mean + SD. Arrow heads indicate time points of images i–iii. (**F**) Corresponding time series of fluorescence emission intensities (I) of roGFP2iL at 405 and 488 nm excitation of a representative seedling. A.U. = arbitrary units. (**G**) Indicated time points from (D). N = 5. Different letters indicate statistical differences according to repeated measures ANOVA with Tukey's multiple comparisons test (P < 0.01). P-values: Supplemental Data Set **S10**.

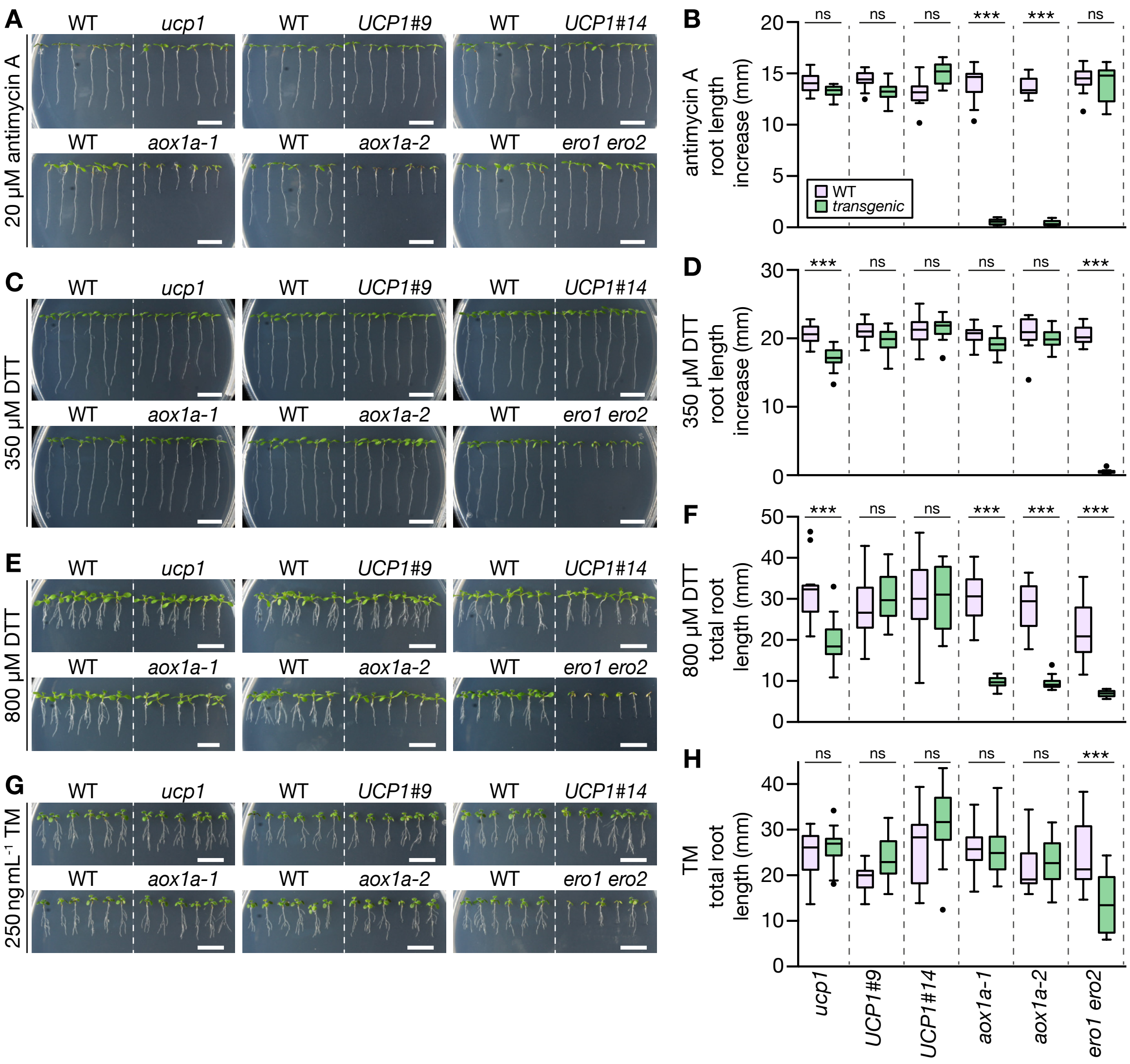

Supplemental Figure S4. Arabidopsis *aox1a* and *ucp1* seedlings show genotype-specific root growth impairments at different DTT concentrations (Supports Figure 3).

Seedling roots of wild-type Col-0 (WT) and transgenic seedlings (*ucp1*: *uncoupling protein 1*, c*UCP1#9* and *#14*: *ucp1* complemented with UCP1 under the control of its native promoter, *aox1a*: *alternative oxidase 1a*, *ero1-3 ero2*: *ER oxidoreductin 1 and* 2). Seedlings were grown vertically on half-strength MS agar plates for 4 d, then transferred to fresh plates supplemented with the treatment chemicals. (**A**) antimycin A; (**C**,**E**) DTT: dithiothreitol; (**G**) TM: tunicamycin. Representative images of seedlings 4 d (A, C) or 6 d (E,G) after transfer. Scale bars: 10 mm. (**B**,**D)** Primary root length increase within 4 d after transfer on new plates. (B) *N* = 18, (D) *N* = 24. (**F**,**H**) Total root length measured 6 d after transfer. (F) *N* = 16–18, (H) *N* = 15–18. (B,D,F,H) Boxplot: 1st and 3rd quartiles with median and Tukey whiskers. Differences were tested after log-transformation of data to establish normal distribution by one-way ANOVA with Bonferroni's multiple comparisons test ($^{ns}P > 0.05$, $^{***}P < 0.001$). *P*-values: Supplemental Data Set **S11**.

Note that *aox1a* primary root length increase was comparable to WT at 350 µM DTT, while that of *ucp1* was significantly reduced by 14% (C,D). Primary root growth was abolished in all lines at 800 µM DTT (E,F), followed by the emergence of lateral roots. Lateral roots were suppressed in *aox1a* and to a lesser extent also in *ucp1*.

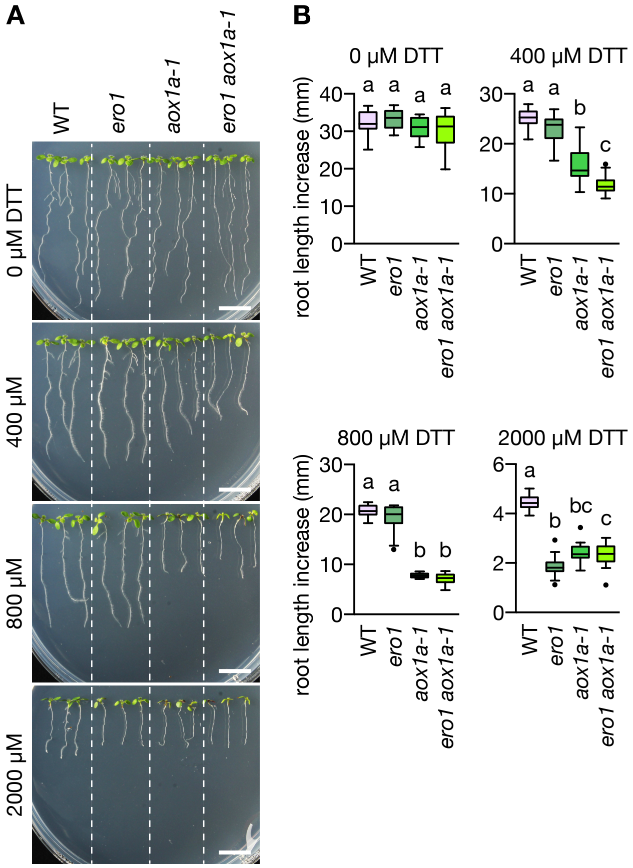

Supplemental Figure S5. Experimental replicate on primary root length increase of WT and transgenic seedlings (*ER oxidoreductin 1; ero1*, *alternative oxidase 1a; aox1a*, *ero1 aox1a-1*) (Supports Figure 4).

(**A**) Seedlings were grown vertically on half-strength MS agar plates for 4 d, then transferred to fresh plates supplemented with 0–2000 µM DTT. Representative images of seedlings 4 d after transfer. Scale bars: 10 mm. (**B**) Root length increase measured 4 d after transfer. 0 µM: N = 14–15, 400 µM: N = 15, 800 µM: N = 15, 2000 µM: N = 15. Boxplot: 1st and 3rd quartiles with median and Tukey whiskers. Differences were tested after log-transformation of data to establish normal distribution. Different letters indicate significant differences (one-way ANOVA with Bonferroni's multiple comparisons test; $P < 0.01$). P-values: Supplemental Data Set **S12**.

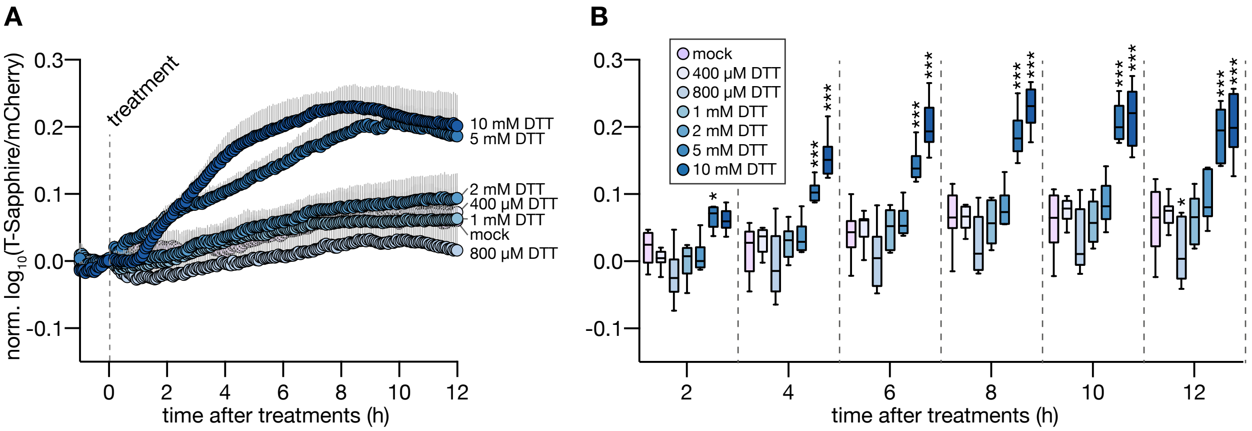

Supplemental Figure S6. *In vivo* monitoring of the cytosolic NAD redox state reveals increased NAD reduction following DTT exposure (Supports Figure 5).

(**A**) Time series of 4-d-old wild-type Col-0 seedlings expressing cytosolic Peredox-mCherry. T-Sapphire and mCherry fluorescence intensities were recorded from seedlings in assay medium in a plate reader and the autofluorescence from corresponding wild-type controls without sensor were used for subtraction. T-Sapphire: excited at 400 ± 5 nm, emission collected at 530 ± 5 nm; mCherry: excited at 540 ± 10 nm, emission collected at 615 ± 9 nm. Dashed line indicates addition of mock or dithiothreitol (DTT). High T-Sapphire/mCherry emission ratios indicate a more reduced NAD pool. *N* = 6–8. Mean + SD. (**B**) Indicated time points from (A). *N* = 6–8. Boxplots: 1st and 3rd quartiles with median and Tukey whiskers. Significant differences compared to mock treatment (C) or WT (E,F) according to two-way ANOVA with Dunnett's multiple comparisons test ($^{ns}P > 0.05$, $^{*}P < 0.05$, $^{***}P < 0.001$). *P*-values: Supplemental Data Set **S13**.

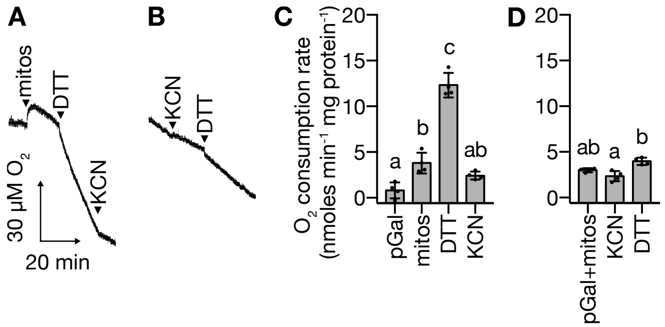

Supplemental Figure S7. DTT induces low rates of oxygen consumption in the presence of isolated mitochondria (Supports Figure 6).

Representative polarographic traces from oxygen consumption assays with purified mitochondria from 14-d-old wild-type Col-0 Arabidopsis seedlings. Arrow heads indicate additions of mitochondria or reagents. (**A**) Incubation buffer supplemented with 0.2 mM propylgallate (pGal). Sequential addition of mitochondria (mitos), 10 mM dithiothreitol (DTT), 1 mM potassium cyanide (KCN). (**B**) Incubation buffer supplemented with 0.2 mM pGal and mitos. Sequential addition of 1 mM KCN, 10 mM DTT. (**C**,**D**) Corresponding oxygen consumption rates from (A) and (B). Mean ± SD. *N* = 4. Different letters indicate significant differences according to repeated measures ANOVA with Tukey's multiple comparisons test ($P < 0.01$). *P*-values: Supplemental Data Set **14**.
Note that pGal was added in a large volume due to its low solubility, resulting in notable oxygen increase (Figure **6A**). To control for potential artefacts, pGal was added to the incubation medium prior to the recordings (A,B).

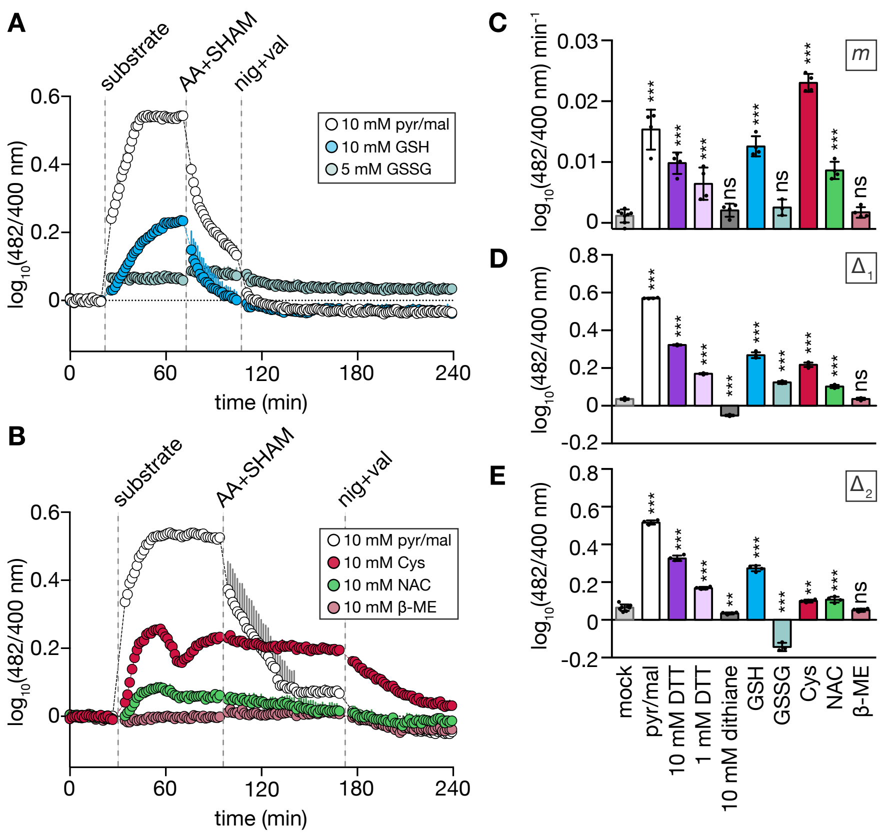

Supplemental Figure S8. Electrons from small thiol molecules can serve as respiratory substrate to induce ΔpH in isolated mitochondria (Supports Figure 6).

(**A**,**B**) pH dynamics in the mitochondrial matrix in response to different substrates recorded via mitochondrial matrix-localised circularly-permuted Yellow Fluorescent Protein (cpYFP) as described in Figure 6D. cpYFP fluorescence ratio depicted as deviation from mean of mock treatment (dotted line at Y = 0). Increase or decrease of ratio indicate more alkaline or more acidic pH, respectively. (A) *N* = 3, (B) *N* = 4. Mean + SD. Vertical dashed lines indicate additions of substrates (β-ME: β-mercaptoethanol, Cys: cysteine, GSH: reduced glutathione, GSSG: glutathione disulfide, NAC: N-acetyl-cysteine, pyr/mal: 10 mM pyruvate + 10 mM malate + 0.3 mM NAD + 0.1 mM thiamine pyrophosphate), mETC inhibitors (AA: 20 µM antimycin A, SHAM: 2 mM salicylhydroxamic acid) and uncouplers (nig: 50 µM nigericin, val: 10 µM valinomycin). Note that pyr/mal trace serves as reference and is identical to pyr/mal in Figure 6E. (**C–E**) Corresponding *m* (slope), Δ_1 (substrate-induced pH difference) and Δ_2 (mETC inhibitor-induced pH difference) from (A,B) and Figure 6E. *N* = 3–8. Mean ± SD. Significant differences to mock according to one-way ANOVA with Dunnett's multiple comparisons test ($^{ns}P > 0.05$, $^{**}P < 0.01$, $^{***}P < 0.001$). *P*-values: Supplemental Data Set **S15**.

Note that mitochondria supplemented with Cys or NAC were largely irresponsive to mETC inhibitor additions, but responsive to the addition of membrane uncouplers (B,C–E). These may be accounted for by mETC self-inhibition due to toxic Cys/NAC degradation products such as H_2S (Dorman et al., 2002).

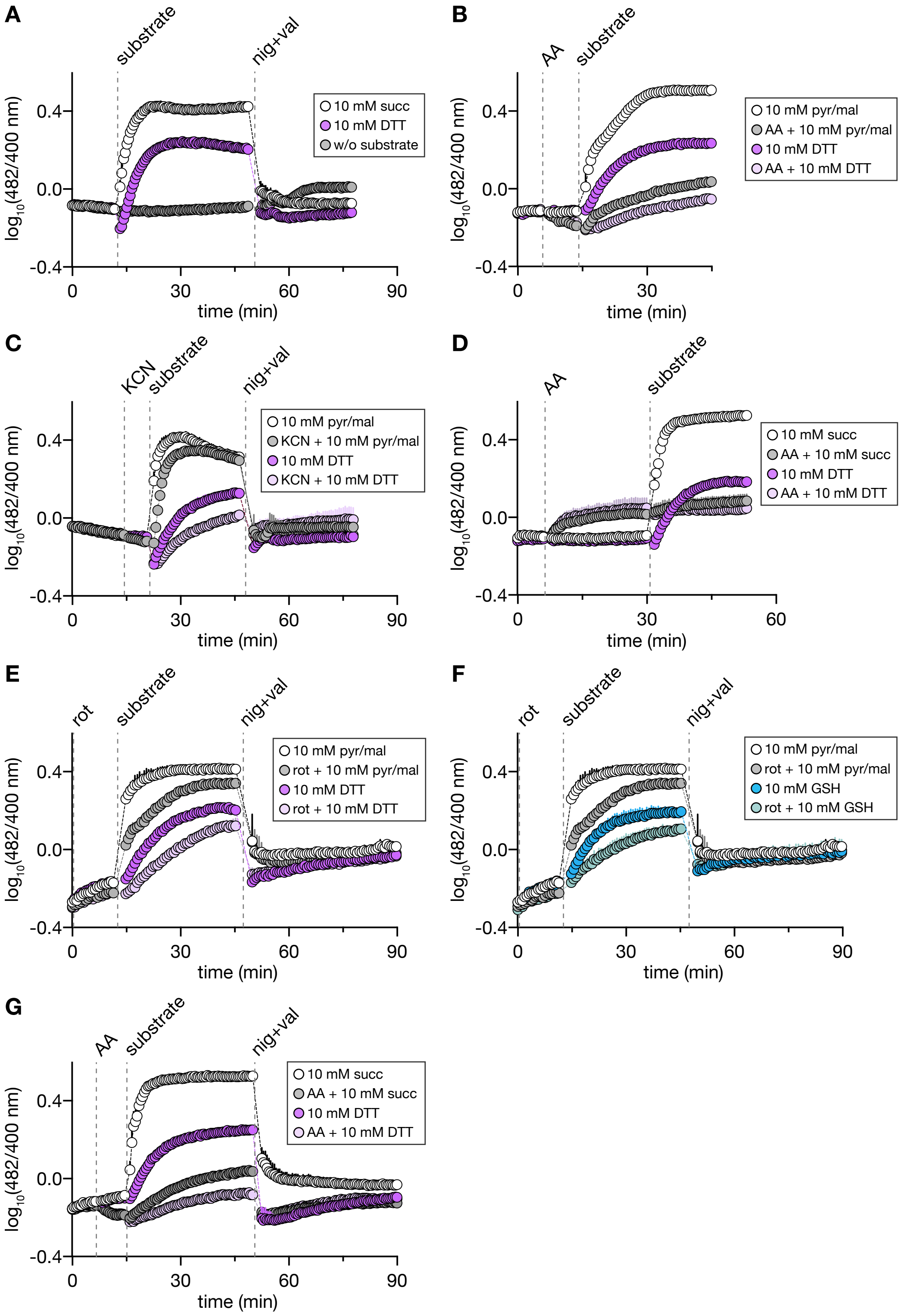

Supplemental Figure S9. Independent replicates confirming mETC-dependent induction of ΔpH in isolated mitochondria by small thiol molecules (Supports Figure 6).

(**A**,**B**) pH dynamics in the mitochondrial matrix in response to different substrates recorded via mitochondrial matrix-localised cpYFP as described in Figure 6D. Increase or decrease of ratio indicate more alkaline or more acidic pH, respectively. (A) *N* = 4, (B) *N* = 3, (C) *N* = 3–4, (D) *N* = 3, (E) *N* = 3, (F) *N* = 3, (G) *N* = 3. Mean + SD. Vertical dashed lines indicate additions of substrates (DTT: dithiothreitol, GSH: glutathione, pyr/mal: 10 mM pyruvate + 10 mM malate + 0.3 mM NAD + 0.1 mM thiamine pyrophosphate, succ: 10 mM succinate + 250 µM ATP), mETC inhibitors (AA: 20 µM antimycin A, KCN: potassium cyanide, rot: rotenone) and ionophores for uncoupling (nig: 50 µM nigericin, val: 10 µM valinomycin). Note that “succ” and “AA + succ” traces serve as references and are identical in E and F.

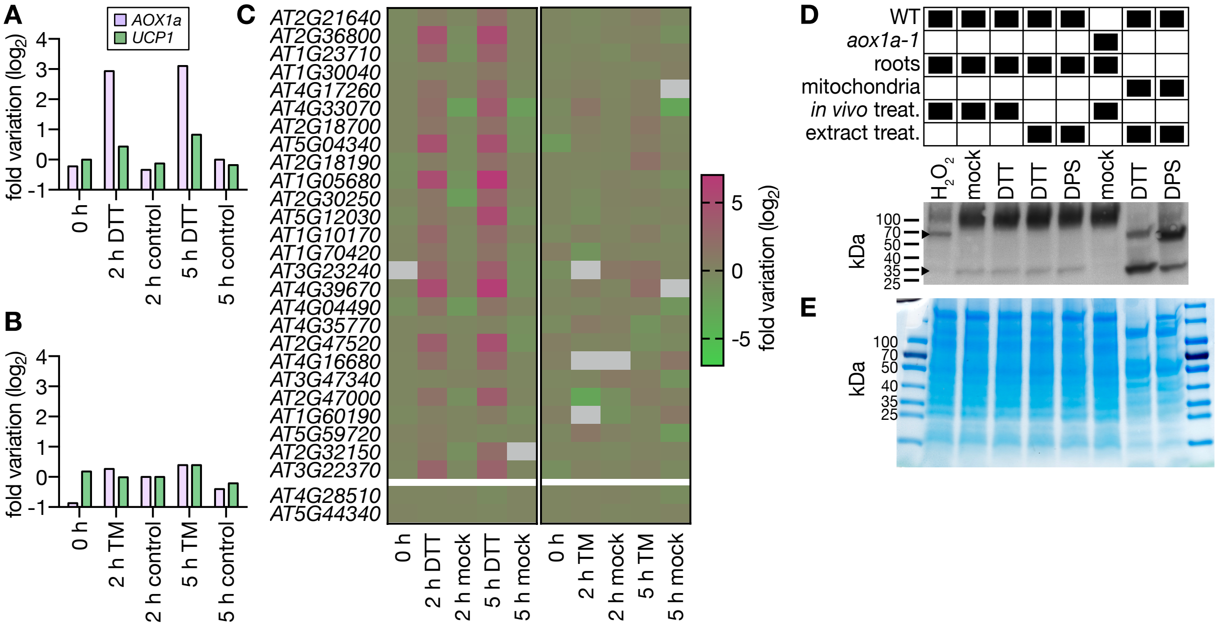

Supplemental Figure S10. Exposure to DTT causes induction of ANAC017-regulated genes, while AOX1a protein shows no redox changes at DTT treatment *in vivo* (Supports Figure 7).

Fold change of *ALTERNATIVE OXIDASE 1a* (*AOX1a*) and *UNCOUPLING PROTEIN 1* (*UCP1*) expression by microarray from 6-d-old Arabidopsis seedlings incubated in treatment solutions only (control) or supplemented with (**A**) 10 mM dithiothreitol (DTT) or (**B**) 5 µg mL^{-1} tunicamycin (TM) for 0, 2, or 5 h. Microarray data retrieved from Martínez and Chrispeels (2003). (**C**) Expression fold changes of genes induced by AA treatments and positively regulated through ANAC017 function (Ng et al., 2013a). Out of the 100 genes revealing the highest AA-induced expression increase (Ng et al., 2013a), microarray elements for 26 candidates were reliably detected in Martinez and Chrispeels (2003). Grey cells: empty values. Bottom: ER stress-insensitive *AT4G28510* (*PROHIBTIN1*) and *AT5G44340* (*TUBULIN BETA CHAIN4*). The fold change in mRNA abundance is based on the average difference (expression intensities) of all probe sets for each condition in the two treatments (DTT and tunicamycin) as presented by Martínez and Chrispeels (2003). (**D**) Immunoblot with AOX1/2 antibody (Agrisera). 18-d-old wild-type (WT) or *aox1a-1* seedlings were treated with 2 mM DTT or 2 mM H_2O_2 for 2.5 h (*in vivo* treatment). Proteins were isolated from roots only and treated with 20 mM N-ethylmaleimide (NEM) to block sulfhydryls to prevent post-extraction disulfide bond formation. NEM-blocking efficiency was validated by treatment of the protein extract with the oxidant dipyridyl disulfide (DPS) and compared DTT (extract treatment). Protein extract from isolated Arabidopsis seedling mitochondria was used as control for antibody specificity. Mitochondria were treated for 10 min in an ultrasonic bath and subsequently treated with 2 mM DTT or 2 mM DPS (without prior NEM blocking). 25 µg protein were loaded per lane. Arrow heads mark reduced and oxidised isoforms, expected at sizes between 30–34 kDa and 60–71 kDa, respectively. (**E**) Corresponding Coomassie-stained gel obtained by non-reducing SDS-PAGE.

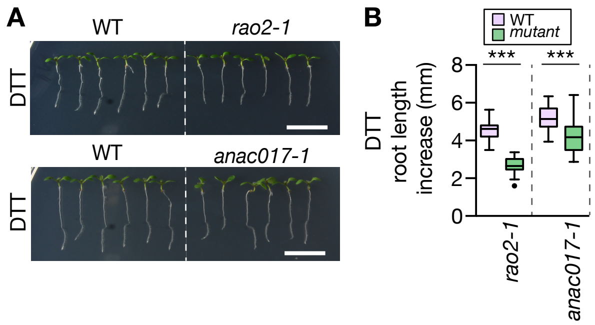

Supplemental Figure S11. ANAC017-dependend signaling is also required to mediate tolerance of root growth to high DTT concentration (Supports Figure 7).

(**A**) Primary root length increase of Arabidopsis wild-type Col-0 (WT), *regulators of alternative oxidase1a* (*rao2-1*) and A*rabidopsis NAC domain-containing protein17* (*anac017-1*) mutant seedlings. Seedlings were grown vertically on half-strength MS agar plates for 4 d, then transferred to fresh plates supplemented with 800 µM DTT. Representative images of seedlings 4 d after transfer. Scale bars: 10 mm. (**B**). Primary root growth within 4 d after transfer on new plates. *N* = 18. Boxplot: 1st and 3rd quartiles with median and Tukey whiskers. Differences were tested after log-transformation of data to establish normal distribution by one-way ANOVA with Bonferroni's multiple comparisons test (****P* < 0.001). *P*-values: Supplemental Data Set **S16**.

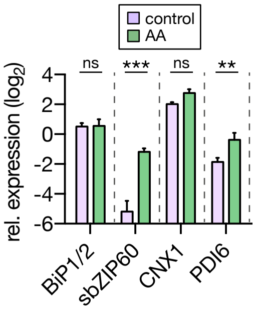

Supplemental Figure S12. mETC inhibition induces ER stress markers (Supports Figure 4).

ER stress marker transcript quantification by RT-qPCR from roots of 12-d-old Arabidopsis wild-type Col-0 seedlings submerged in treatment solutions without (control) or with 50 µM antimycin A (AA) for 2.5 h. Transcripts: *BINDING PROTEIN 1* and *2* (*BiP1/2*), *spliced BASIC LEUCINE ZIPPER 60* (*sbZIP60*), *CALNEXIN 1* (*CNX1*), *PROTEIN DISULFIDE ISOMERASE 6* (*PDI6*). Mean + SD. *N* = 3 plates with pooled roots from 18–20 seedlings each. Note that the data are based on the dataset shown in Figure 4A. Differences were tested by two-way ANOVA with Bonferroni's multiple comparisons test ($^{ns}P > 0.05$, $^{**}P < 0.01$, $^{***}P < 0.001$). *P*-values: Supplemental Data Set **S17.**

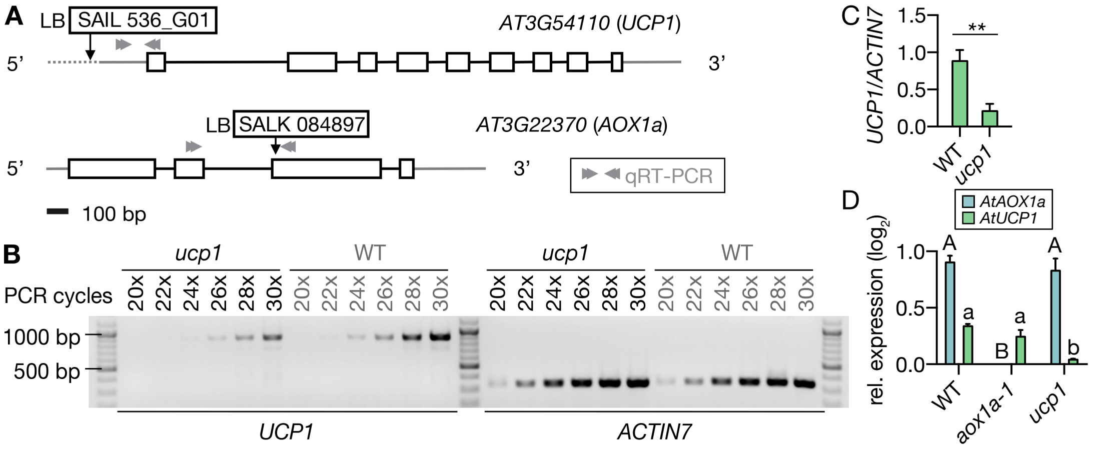

Supplemental Figure S13. Molecular characterisation of *ucp1* (Supports Figure 3).

(**A**) Gene models of *Arabidopsis thaliana UNCOUPLING PROTEIN 1* (*UCP1*) and *ALTERNATIVE OXIDASE 1a* (*AOX1a*). Boxes: exons, black lines: introns, grey lines at 5’ and 3’ end: UTRs, dotted grey line: un-transcribed sequence, grey double arrow heads: RT-qPCR primers used in (C). Name and orientation of T-DNA insertions are indicated on top of corresponding genes. (**B**) Agarose gel image of semi-RT-qPCR with primers amplifying full-length gene transcripts from cDNA reverse transcribed from RNA isolated from pooled 7-d-old seedlings. (**C**) Quantification of *UCP1* and *ACTIN7* bands from semi-RT-qPCR shown in (D). Y-axis: Measured band intensities shown as ratio from $UCP1_{26x}/ACTIN7_{20x}$, $UCP1_{28x}/ACTIN7_{22x}$ and $UCP1_{30x}/ACTIN7_{24x}$. Significant difference between genotypes according to two-sided t-test (***P* < 0.001). (**D**) Transcript quantification by RT-qPCR on cDNA reversely transcribed from RNA isolated from leaves of 3-week-old Arabidopsis plants grown on soil under standard conditions. Reference gene: *SAND FAMILY PROTEIN. N* = 3 biological replicates. Different letters indicate significant differences according to two-way ANOVA with Tukey's multiple comparisons test (*P* < 0.01). *P*-values: Supplemental Data Set **S18**.

Supplemental Table S1. List of primers.

primer ID	gene	gene ID	orientation	sequence	purpose
4672	*sbZIP60*	*AT1G42990*	fw	CGATGATGCTGTGGCTAAAA	RT-qPCR
4673	*sbZIP60*	*AT1G42990*	rv	TCAGCAACGAAGCACTCAAG	RT-qPCR
4664	*BiP1/2*	*AT5G28540*	fw	CCACCGGCCCCAAGAG	RT-qPCR
4665	*BiP1/2*	*AT5G28540*	rv	GGCGTCCACTTCGAATGTG	RT-qPCR
4656	*CNX1*	*AT5G61790*	fw	CGCTGGATCGTTTCGAAGAA	RT-qPCR
4657	*CNX1*	*AT5G61790*	rv	CACACTCAAGTCCTTCCTGGAA	RT-qPCR
3892	*TIP41*	*AT4G34270*	fw	AATGCGTTTGACGCACTAGC	RT-qPCR
3893	*TIP41*	*AT4G34270*	rv	GAGACGGCTTGCTCCTGAAT	RT-qPCR
4680	*PDI6*	*AT1G77510*	fw	AAGTGGTCCTGCTTCTGTTGAA	RT-qPCR
4681	*PDI6*	*AT1G77510*	rv	TTGAACAGCCTCACTGCAGGT	RT-qPCR
3917	*AOX1a*	*AT3G22370*	fw	TCGTTGGCCTACCGATTTGT	RT-qPCR
3918	*AOX1a*	*AT3G22370*	rv	ACCATTCCAGGTACTGCTGC	RT-qPCR
3913	*UCP1*	*AT3G54110*	fw	TAGCCGTAATCGTCGTCGTC	RT-qPCR
3914	*UCP1*	*AT3G54110*	rv	GGGCAAGGAAAGGTCGGATT	RT-qPCR
4688	*ACT8*	*AT1G49240*	fw	TCAGCACTTTCCAGCAGATG	RT-qPCR
4689	*ACT8*	*AT1G49240*	rv	ATGCCTGGACCTGCTTCAT	RT-qPCR
P19	*SAND*	*AT2G28390*	fw	CCATATTGCAAGAAGTTTGCGCGTCTG	RT-qPCR
P20	*SAND*	*AT2G28390*	rw	GCAAGTCATCGGGATGGAGAGACG	RT-qPCR
P23	*UCP1*	*AT3G54110*	fw	ATGGCCGATGGTGAGGA	semi-qRT-PCR
P24	*UCP1*	*AT3G54110*	rv	GAAGCATTTCCTGTGAACAATC	semi-qRT-PCR
P25	*ACT7*	*AT5G09810*	fw	ATGGTGGCGGCTGGTAA	semi-qRT-PCR
P26	*ACT7*	*AT5G09810*	rv	CTTTTGGACGCATCGAGTTC	semi-qRT-PCR
