# Supplementary material for: Reductive stress triggers ANAC017-mediated retrograde signaling to safeguard the endoplasmic reticulum by boosting mitochondrial respiratory capacity
Source: Plant Cell. 2022 Jan 25;34(4):1375–95. doi: 10.1093/plcell/koac017 (PMC9125394; doi:10.1093/plcell/koac017)
Supplement: koac017_Supplementary_Data [file TPC2021-RA-00408R2 Peer Review Report.pdf › TPC2021-RA-00408R2 Peer Review Report.pdf]

Reductive stress triggers ANAC017-mediated retrograde signaling to safeguard the endoplasmic reticulum by boosting mitochondrial respiratory capacity

Philippe Fuchs, Finja Bohle, Sophie Lichtenauer, José Manuel Ugalde, Elias Feitosa Araujo, Berivan Mansuroglu, Cristina Ruberti, Stephan Wagner, Stefanie J. Müller-Schüssele, Andreas J. Meyer, Markus Schwarzländer

Corresponding author: Markus Schwarzländer markus.schwarzlander@uni-muenster

Review timeline:

**TPC2021-RA-00408**	Submission received:	May 13, 2021
	1st Decision:	June 30, 2021 *revision requested*
**TPC2021-RA-00408R1**	1st Revision received:	Oct. 21, 2021
	2nd Decision:	Dec. 1, 2021 *accept with minor revision*
**TPC2021-RA-00408R2**	2nd Revision received:	Dec. 16, 2021
	3rd Decision:	Dec. 18, 2021 *acceptance pending, sent to science editor*
	Final acceptance:	Jan. 14, 2022

REPORT: (The report shows the major requests for revision and author responses. Minor comments for revision and miscellaneous correspondence are not included. The original format may not be reflected in this compilation, but the reviewer comments and author responses are not edited, except to correct minor typographical or spelling errors that could be a source of ambiguity.)

TPC2021-RA-00408 1st Editorial decision – *revision requested* **June 30, 2021**

The editorial board agrees that the work you describe is substantive, falls within the scope of the journal, and may become acceptable for publication, pending revision and potential re-review.

In post-review discussion, it was agreed that this work is exciting and likely to be really impactful. However, this excitement also presents the imperative to fully validate and strengthen your arguments. There are three main points where we feel revision and likely additional experimentation would be needed: 1) additional validation of the argument that thiols can be donors for the mETC, which is a surprising result, perhaps using alternative substrates; 2) addressing issues of reproducibility between experiments; 3) additional validation of the ER link. The reviews, appended below, provide a number of experiments that would address each of these points. It was agreed that experiments with NDB2 knockouts would strengthen your argument even further, but that they would not be required if you were able to provide more convincing recordings with the current material.

Please don't hesitate to contact us with questions or for clarification; or if you need more than the typical 60 days to complete this work.

--- Reviewer comments:

Reviewer #1 (Comments for the Author):

The manuscript addresses the long standing question of the role of AOX in mediating electron flux in mitochondria. Starting from the surprising observation of reduced root growth of AOX1 and the known AOX1a gene induction in response to inhibition of mitochondrial electron transport, the authors assemble a convincing line of experiments. They hypothesize that excess reduction capacity not only affects ER function with respect to protein folding but leads to over-reduction of mETC and concomitant induction of AOX1a expression which in the helps to alleviated reductive stress. They show that he latter is at least in part mediated by the known ANA017 transcription factor. Along the way the authors contribute important observations for the field, such as the significant electron transfer from reduced thiols into the mETC and further evidence for a mitigating function of mitochondrial UCP.

The experimental design of the different approaches is convincing and the manuscript is presented in a very clear and straight forward way. The difficulty in such cell physiological hypotheses are the functional links and proofs rather than correlations because often genetic approaches can have highly pleiotropic consequences. Indeed, some of the data of correlative nature and often based on gradual effects. However, these instances are clearly indicated by the authors and where possible complemented by direct evidence such as live cell imaging of mitochondrial activity towards thiols.

Only a few aspects need further clarification. Among them, the reader wonders why none of the other AOX genes was investigated (just to be sure that only AOX1a expression is responding to treatments).

Glutathione was shown to be a suitable donor for mETC, but does operate at appreciable rates during regular growth on cost of the red/ox glutathione ratio?

The section on the response of the ER to reductive stress dependency on mitochondrial respiration flexibility is crucial for the development of the hypothesis and to show that the ER is specifically affected. Yet this part relies entirely on quantitative expression changes of marker genes in aox1a and ucp1 seedlings and not physiological data.

How can one demonstrate that those mutants really 'suffer' more from DTT induced reducing stress? An independent and more direct line of evidence rather than the approaches described in the manuscript is required to finally proof that mitochondrial respiration safeguards the ER from reductive stress. Is there any evidence from research in yeast?

Reviewer #2 (Comments for the Author):

The paper describes a very detailed study using an impressive array of sensing and other techniques to investigate the effect of exogenous reduced thiols on seedling growth, respiration and ER function. The experiments have been carefully carried out for the main and I find most of the data convincing, but the authors should address the following comments

1. Some of the root responses to external stimuli are rather variable and this should be explained - for example aox1a and aox1a-2 mutations effect on root response to DTT in figure 1 is substantially greater than in figure2.

2. I am not convinced that DTT is an effective respiratory substrate per se, although it clearly affects components of the mETC. The oxygen electrode data in Figure 6 are not convincing, especially the responses to respiratory inhibitors. I have added DTT to isolated mitochondria in many such experiments over the years and have not seen significant direct effects on oxygen uptake by mitochondria. This makes it unlikely, in my opinion, that reduced thiols will themselves act as respiratory substrates in vivo; rather, as the authors discuss, they are likely to affect respiration via NAD(P)H levels in the cytosol (in addition to stimulating AOX synthesis and activation). This could be tested using the publicly available ndb2 Arabidopsis KO mutants, since NDB2 is the major external NADH dehydrogenase in mitochondria. I think it is premature to say that reduced thiols act as respiratory substrates, and this statement in the abstract should be omitted.

3. I think it should be pointed out that DTT not only induces AOX synthesis (or at least transcripts) but that it is also likely to ensure its activation by reducing the conserved Cys-residues of the enzyme. In this context, I would like to see some western blots of AOX in root or leaf extracts with and without added reducing agents.

Reviewer #3 (Comments for the Author):

The manuscript by Fuchs et al provides evidence for a role of mitochondrial respiration to safeguard the ER from reductive stress. The manuscript is highly interesting, especially the possibility of alternative reductants feeding into mitochondrial respiration, but also in its use of novel biosensors, and has potential to attribute new function to mitochondrial stress responses. I have however a few concerns:

Major revisions:

General comment:

DTT has a very high reductive potential and therefore will have major impact on thiol balance, protein function/structure and other cellular components, i.e. it likely has a substantial pleiotropic effect. Although I agree that

it can be used to identify genes/proteins involved in the response to such stress for further characterisation, as has been done for the UPRER, I find it much harder to attribute physiological responses to a specific process here. In my opinion the authors do not account sufficiently for such pleiotropic effects, e.g. by metabolic or transcriptomic experiments, to establish the extent of changes on a more global scale. Even a few targeted measurements, e.g. the concentrations/ratios of GSH/GSSG, ascorbate/dehydroascorbate and a few stress-markers by qRT-PCRs would shed more light on this. This would establish a baseline to interpret results on mitochondria and the ER more carefully. The authors rather reduce the DTT response to mitochondria-ER interactions without accounting for independent influences from other compartments, i.e. cytosol or chloroplasts, that may run in parallel.

L144-148

Although I understand that DTT is somewhat difficult to work with, it is unclear to me how the apparent variable effect of DTT has been dealt with across experiment replicates. This somewhat lends itself to picking favourable outcomes, while discarding the unexpected ones, as an optimisation strategy? The differences in phenotypes of plants in Figure 1 and Supplemental Figure 3 for e.g. 800µM are quite substantial and suggest a two-fold variation of DTT can lead to the same result. I would assume that using fresh solutions each time would ameliorate these problems. Can the authors provide some clarification on this? Also, in some experiments different concentrations or submergence (up to 10mM DTT) have been used, which makes the comparison across experiments difficult.

L148:

The authors claim their DTT treatment has no phenotypic effect on leaf tissues, but provide no evidence for this. Again, in Supplemental Figure 7 the leaves look much bigger than for seedlings in Figure 1. Also, in leaf disc assays (Figure 5) the authors do find an effect of DTT treatment on green tissues. Some evidence should be presented that DTT does not have a general effect that impacts photosynthesis with a knock-on effect on (root) growth.

L183:

The authors provide no further information for the ero1 ero2 line. It is therefore impossible to assess any results presented for this line. The authors need to decide to either remove this line from the manuscript or provide the necessary information on its generation, confirmation, characterisation etc.

L203/211-212:

Is there any reason that the ucp1 line has this substantial variation and this is reproducible ? This goes back to my earlier point on the selective inclusion of results based on phenotype (L144-148, see above). Also, as only two DTT concentrations are given it is hard to deduce 'optima'.

L285-302

Although I like the use of biosensors, I feel direct measurement of respiration and including such data in a figure of the main manuscript is important. Although DTT is a tool, providing solid evidence for endogenous metabolites is crucial to support a physiological relevance. I know the latter is a somewhat stale argument, but I believe this is an important point for the story.

L304-324

Although presented results clearly show that ANAC017 is involved in the stress response, I am not certain if the authors can ensure this is not a response to a pleiotropic effect unrelated to the ER. A comparison with TM treatment would allow to better differentiate. This might also show if mitochondrial stress impacts the ER, or if the ER stress impacts mitochondrial signalling. Also, increased expression of AOX1a in an ire1a ire1b double mutant (Ng et al 2013), and increased expression of bZIP60 under AA treatment and in ANAC017 overexpressing lines (Meng et al 2019) already suggest a pathway from mitochondria to the ER. This is also missing from the discussion.

Minor revisions:

The discussion is quite long (longer than the results) and could be more concise. But known connections between mitochondria and the ER, e.g. via ANAC017, bZIP60 (see above) should be discussed.

L153-154:

Have the authors done concentration series for those stresses? Otherwise this needs to be phrased more carefully, as other concentrations might have an effect.

L548

Should be Supp Figure 6.

I believe there is something wrong with the presentation of data. The scale is not centre around zero, I am unsure what the 0h timepoint would be calculated against, i.e. fold change against what?

Figure 2:

I am not sure if the time points in A and B should match, but I couldn't follow what was given in the legend, e.g. there is nothing marked in grey in A. Please check.

Suppl Figure 7 and 8: No reference anywhere in the text.

TPC2021-RA-00408R1 1st Revision received October 21, 2021

Reviewer comments and **author responses**:

EDITOR: In post-review discussion, it was agreed that this work is exciting and likely to be really impactful. However, this excitement also presents the imperative to fully validate and strengthen your arguments. There are three main points where we feel revision and likely additional experimentation would be needed:

1) additional validation of the argument that thiols can be donors for the mETC, which is a surprising result, perhaps using alternative substrates;

We are aware that we appear to make an extraordinary claim and that generally requires extraordinary evidence. We provide that evidence through three independent experimental approaches to scrutinize our working model. However, the claim is not completely out of the box at second glance since there is a plausible rationale why thiol-based provision of electrons to the respiratory chain has not been picked up in oxygen electrode experiments before. Further there are well documented biochemical mechanisms for electron provision to the mitochondrial electron transport chain by thiols (even specifically DTT), including:

- **DTT can reduce human ubiquinone in vitro (Kishikawa et al., 2011)**
- **DTT can reduce *E. coli* ubiquinone (Meyrat and Ballmoos, 2019)**
- **DTT can reduce Mia40, which reduces Erv1, which reduces cytochrome *c* of mETC (Bihlmaier et al., 2007; Peleh et al., 2017)**

In the light of the recorded data and despite our best attempts to scrutinize our data we find it hard to see any plausible alternative interpretation.

To address the concerns about the concept of thiols as electrons donors for the mETC, we have now included additional measurements (Suppl. Figure 8). The data show the coherence and reproducibility of DTT, and of GSH as an endogenous thiol, as electron donors to the respiratory chain and included additional controls with respiratory inhibitors. As such they provide further weight to the conclusion.

We further clarify a potential misunderstanding with reviewer #2 emphasizing that we do not propose that thiols are directly oxidized by mETC proteins or complexes but are rather likely to be oxidized by other cellular systems that then transfer the electrons to the mETC.

Following this line of argument, we now explore the possibility of NAD reduction further. For that purpose, we replicated the NAD redox biosensing measurements at acute DTT expose in young seedlings, assessed the concentration dependency of the NAD redox response and included the different genetic backgrounds (Figure 5; Suppl. Figure 5). Strikingly we observed that aox1a shows more pronounced NAD reduction in response to DTT than the WT, which provides independent evidence for an NAD-de-pendent link between thiols and the mETC.

We further include a Western blot analysis to validate the redox response of AOX1a to the DTT treatment (Suppl. Figure 9D,E), which confirms that the redox-active cysteines are fully reduced in the roots and are not further reduced by DTT.

Data added:

- **Figure 5**
- **Suppl. Figure 6**
- **Suppl. Figure 9**
- **Suppl. Figure 10 D,E**

2) addressing issues of reproducibility between experiments;

We now clarify the experimental design that we had carefully optimized to account for the intrinsic instability of DTT. We now point out more specifically the difference between (a) effectiveness of the DTT treatment overall (as a prerequisite for differential growth between genotypes), and (b) the actual differential root growth effects between WT and mutant lines. To provide a better overview of both (a) and (b), we have added a dataset to exemplify the dose response to DTT (Suppl. Figure 2). The data body clearly shows the strict reproducibility of the phenotype at the qualitative level, i.e. stronger impairment of root growth in the *aox1a* or *ucp1* line as com-pared to their corresponding control in the presence of DTT in the high µM range. They also illustrate the sources of quantitative variability (i.e. absolute DTT concentration for the differential responses to arise; relative degree of inhibition in *aox1a* lines com-pared to the *ucp1* line).

For more details we refer to our responses to reviewer #1 and #3. We added a dose-response dataset to the manuscript (Suppl. Figure 2)

Data added:

- **Suppl. Figure 2**

3) additional validation of the ER link.

OUR RESPONSE: We consider this point as particularly central and are very grateful for the feedback. We have been extremely grateful for the very kind extension of the re-submission deadline, which has allowed us to generate an *aox1a ero1* double mutant line, in which the respective oxidase capacities in both the mitochondria and the ER are weakened. Root growth of seedlings of this line shows synergistic sensitivity DTT, as compared to the single mutants, providing compelling and orthogonal evidence for the ER link (Figure 4B-D; Suppl. Figure 5).

Further, we validate the model that the ER is particularly prone to DTT-mediated reductive stress. We used five different Arabidopsis lines that each express a glutathione redox biosensor of identical midpoint potential (roGFP2) in a different subcellular compartment (cytosol/nucleus, peroxisomes, plastid stroma, mitochondrial matrix, ER lumen). We challenge seedlings of those lines with different DTT treatments to assess their relative response (Suppl. Figure 3A). Those data show that the glutathione pool in the ER is much more oxidized, whereas the glutathione pools of the other compartments are highly reduced. Consequently, the ER responds particularly sensitively to DTT. This is entirely expected based on previous work and the chemical reaction mechanism of DTT with endogenous Cys-based compounds. Yet, the data provide empirical validation for the focus on ER stress in the specific experimental system.

Data added:

- **Figure 4B-D**
- **Suppl. Figure 3A**
- **Suppl. Figure 5**

The reviews, appended below, provide a number of experiments that would address each of these points. It was agreed that experiments with NDB2 knockouts would strengthen your argument even further, but that they would not be required if you were able to provide more convincing recordings with the current material.

We provide a point by point response to the reviews and have made best use of the feedback to address the points

through additional experiments and textual adjustments.

We hope that the additional evidence that we provide based on the current lines, as well as the addition of the *aox1a ero1* double mutant will be satisfactory in the context of the current manuscript.

ADDITION OF MOLECULAR DATA ON THE *ucp1* LINE:

As part of the molecular validation of the *ucp1-1* background we mapped the T-DNA insertion site more upstream than previously reported (Sweetlove et al., 2006), i.e. in the promoter region of the UCP1 locus (Supplemental Figure 13A). Our subsequent assessment of the transcript levels revealed that the full length UCP1 transcript was reduced by at least 80%, but still detectable (Supplemental Figure 13B-D). Hence, *ucp1-1* represents a knockdown, rather than a knockout line, which is consistent with the Western blotting analysis for UCP1 protein abundance reported previously (Sweetlove et al., 2006). Then the remaining weak band was interpreted as cross-reactivity of the antibody with other DIC-family proteins.

This finding is important for further use of the *ucp1* line, which is why we would like to document it appropriately and have incorporated the data in Supplemental Figure 13. The rationale of using the line to limit respiratory flexibility in the context of this work is unaffected. It is apparent from the *ucp1* root phenotypes that the decrease in UCP1 leads to a limiting situation. We have updated the Discussion and Methods sections accordingly.

Data added:

- **Suppl. Figure 13**

Response to the Reviewers

We thank all three reviewers for their hugely helpful and thoughtful advice. We are particularly glad for the joint support of our manuscript overall and have done our best to address open points, where possible through additional empirical hypothesis testing. We respond to the comments point-by-point and apologize that our responses to specific points are more extensive. However, we deemed them extremely interesting and although they cannot be sufficiently discussed in their full context here, we have been aiming to provide a realistic representation of our thoughts.

Reviewer #1 (Comments for the Author)

The manuscript addresses the long standing question of the role of AOX in mediating electron flux in mitochondria. Starting from the surprising observation of reduced root growth of AOX1 and the known AOX1a gene induction in response to inhibition of mitochondrial electron transport, the authors assemble a convincing line of experiments. They hypothesize that excess reduction capacity not only affects ER function with respect to protein folding but leads to over-reduction of mETC and concomitant induction of AOX1a expression which in the helps to alleviated reductive stress. They show that he latter is at least in part mediated by the known ANA017 transcription factor. Along the way the authors contribute important observations for the field, such as the significant electron transfer from reduced thiols into the mETC and further evidence for a mitigating function of mitochondrial UCP.

The experimental design of the different approaches is convincing and the manuscript is presented in a very clear and straight forward way. The difficulty in such cell physiological hypotheses are the functional links and proofs rather than correlations because often genetic approaches can have highly pleiotropic consequences. Indeed, some of the data of correlative nature and often based on gradual effects. However, these instances are clearly indicated by the authors and where possible complemented by direct evidence such as live cell imaging of mitochondrial activity towards thiols.

Only a few aspects need further clarification. Among them, the reader wonders why none of the other AOX genes was investigated (just to be sure that only AOX1a expression is responding to treatments).

We agree that the observation of the link between mitochondrial respiration and reductive stress management has the potential to be expanded to additional candidate proteins which may be expected to uncouple the OXPHOS system, including the other AOX isoforms, but also the other UCP isoforms and the alternative NAD(P)H dehydrogenases (NDA/Bs).

In this work we focused on AOX1a (and UCP as a control, that is not related to AOX1a at the protein level but at the

process level), and the choice of model was carefully considered. Based on the available evidence on expression and capacity we would not expect any similar effects in any single knockout line of one of the other AOX isoforms. Even if one of the other AOX isoforms did respond to the reductive stress treatments the observed phenotypes clearly show such a putative response is unable to complement the lack of AOX1a.

The observation that the *aox1a* single mutant turned out sufficient and effective as a means to constrain the flexibility of respiratory capacity, as evidenced by root growth arrest on antimycin A, and the DTT-induced root growth genotype. Limitation of the system by the absence of AOX1a alone is consistent with AOX1a being the most abundant (and stress responsive) AOX isoform in seedlings. It is also consistent with public AOX gene expression data (AOX1b: expressed in flowers; AOX1c: expressed at very low levels; AOX2: expressed in seeds). AOX isoforms other than AOX1a were beyond the detection limit in the Western blotting analysis of seedling roots (using a polyclonal AOX1/2 antibody) that we have now added to Suppl. Figure 10 D,E.

Despite its low expression levels AOX1d may be interesting candidate based on its global expression and its response to absence of AOX1a and mitochondrial impairment. Previous work has established that AOX1d is unable to make a contribution comparable to AOX1a to AOX capacity and its induction in the *aox1a* background was unable to complement AOX1a function (Strodtkötter et al., 2009, Mol Plant). It may be promising to assess an *aox1a aox1d* double mutant following the rationale to limit AOX capacity in seedlings even slightly more than in the *aox1a* single lines. Given that *aox1a* alone is sufficient to achieve functional limitation, however, it offers all required characteristics as a model in the context of this study.

Glutathione was shown to be a suitable donor for mETC, but does operate at appreciable rates during regular growth on cost of the red/ox glutathione ratio?

This is a fascinating question. It is raised by the observation of GSH being able to act as an electron donor to the mETC of isolated mitochondria. We have been discussing the possibility of glutathione being constantly oxidized and re-reduced in vivo with several colleagues with strong expertise in glutathione redox biology. We cannot provide a definitive statement since there is currently no method to measure the in vivo electron fluxes through the glutathione pool (getting such an approach at hand would be a major technical breakthrough in redox biology). Steady oxidation of GSH by a mechanism that involves the mETC as balanced by the high activity of both glutathione reductases (GR1 in the cytosol & peroxisomes, GR2 in the mitochondrial matrix and plastid stroma), at the expense of metabolically-derived NAD(P)H, may constitute an attractive mechanism to rapidly regulate glutathione redox potential (E_{GSH}) in the cell. Such a mechanism is thermodynamically feasible and energetically efficient – and it would allow glutathione oxidation by a mechanism independently from ROS (such as H_2O_2). In such a model the mitochondria would deliver net 'glutathione oxidase' activity. At this stage we do not have sufficient evidence to propose such a model, but we believe that the data from the isolated mitochondria are extremely interesting, which is why we have started preliminary work to follow up on this lead. It may have major implications for GSH-based redox control not just in plants, but across biology.

A first set of preliminary data indicates that the reaction catalyzed by GR is fully reversible when we reconstitute GSH:GSSG ratios/ EGSH values as found in the cytosol in vivo (>50,000 : 1; -320 mV). This is in contrast to the general view that implies that the reaction is irreversible, which is based on assumptions on GSH:GSSG ratios that we know have been systematically underestimated due to the intrinsic limitations of analytical methods (reviewed in Schwarzländer et al., 2016, Antiox Redox Signal). The possibility of NAD(P) and glutathione redox potentials in the being coupled has been discussed also by our colleagues (e.g., Noctor et al., 2012, Plant Cell Environ), and it remains a current, central and unresolved question in redox biology. Our preliminary data indicate that under physiological conditions the NAD(P) pool and the glutathione pools of the cytosol and the mitochondria may be in equilibrium as connected through GR-based catalysis. Such a mechanism may underpin our observation in vivo, but to make more definite statements the arising hypotheses need to be tested experimentally and that goes far beyond the scope of this study.

To gain additional in vivo evidence we have expanded the cytosolic NAD redox measurements using the biosensor Peredox-mCherry and have completely revised Figure 5 (and added a new Suppl. Figure 6) through the measurement of additional datasets. Acute DTT exposure caused NAD reduction in vivo and we made the new and highly interesting observation that the response was stronger in the *aox1a* seedlings. Since glutathione is a major thiol in vivo it may be speculated that it is involved in mediating electron flux from DTT to NAD. We discuss the potential in vivo electron routes in the Discussion section.

The section on the response of the ER to reductive stress dependency on mitochondrial respiration flexibility is crucial for the development of the hypothesis and to show that the ER is specifically affected. Yet this part relies entirely on quantitative expression changes of marker genes in aox1a and *ucp1* seedlings and not physiological data.

How can one demonstrate that those mutants really 'suffer' more from DTT induced reducing stress? An independent and more direct line of evidence rather than the approaches described in the manuscript is required to finally proof that mitochondrial respiration safeguards the ER from reductive stress. Is there any evidence from research in yeast?

We thank the reviewer and agree that any single line of evidence needs independent validation. That said, the specific transcript response of UPRER markers is the established indicator of ER stress across species, including yeast.

Since we share the wish of the reviewer for further scrutiny, however, we have made it a priority of this revision to establish a system to independently test the hypothesis of mitochondrial respiration-dependent ER stress alleviation.

For that purpose, we generated a double mutant by crossing the single lines *aox1a-1* and *ero1*, and selection of homozygous offspring. The rationale behind using this cross was that the *ero1* line has lower ERO capacity in the ER and is hence more sensitive to reductive stress. Since the second ERO isoform (ERO2) is still present in the ER, *ero1* seedlings only show the root growth phenotype, as a physiological indicator of ER stress, under DTT challenge (see separate manuscript on ERO that we provide in the resubmission). We reasoned that this phenotype is NOT expected to be synergistically exacerbated if AOX1a has NO role in alleviating ER stress. The experiment that we devised provides clear and reproducible evidence for a synergistic impact of *ero1* and *aox1a*, however. We have included the data in Figure 4 B-D and Suppl. Figure 5. They validate the working model that limitations in mitochondrial function can contribute to ER stress.

As the reviewer indicates, evidence has been emerging for a role of mitochondria in safeguarding the ER from reductive stress in yeast and mammalian cells. This is particularly exciting, since the results and interpretations were obtained completely independently. Further the specific experimental systems and approaches differ, which may point towards a mechanism that operates not just in plants but more generally in eukaryotes. Yet, the plant model may be particularly informative based on its particularly high flexibility of the respiratory system. We refer to two recent studies specifically in the Discussion section.

Hijazi, I., Knupp, J., and Chang, A. (2020). Retrograde signaling mediates an adaptive survival response to endoplasmic reticulum stress in *Saccharomyces cerevisiae*. J. Cell Sci. 133.

Knupp, J., Arvan, P., and Chang, A. (2019). Increased mitochondrial respiration promotes survival from endoplasmic reticulum stress. Cell Death Differ. 26: 487–501.

Reviewer #2 (Comments for the Author)

The paper describes a very detailed study using an impressive array of sensing and other techniques to investigate the effect of exogenous reduced thiols on seedling growth, respiration and ER function. The experiments have been carefully carried out for the main and I find most of the data convincing, but the authors should address the following comments

1. Some of the root responses to external stimuli are rather variable and this should be explained - for example aox1a and aox1a-2 mutations effect on root response to DTT in figure 1 is substantially greater than in figure2.

We understand this critique, which picks up on the transparent representation of the phenotyping data and are glad that we have a chance to comment. We have spent a large proportion of time and effort to establish the system to be robust as this is critical for the interpretation of the data. Since the study relies on DTT as a tangible model treatment, we not only limited the variability between experiments by following a strict protocol of preparing the media. The issue was further considered in the experimental design. Seedlings of different genotypes were systematically grown side-by-side with WT seedlings as reference on the same plates. This accounted for potential variability between the medium in individual plates. Furthermore, comparisons of root length growth were performed within the same experiment to account for potential variation between experiments. We now point out more specifically the difference between (a) effectiveness of the DTT treatment overall (as a prerequisite for differential growth between genotypes), and (b) the actual differential root growth effects between WT and mutant

lines. The data body clearly shows the strict reproducibility of the phenotype at the qualitative level, i.e. stronger impairment of root growth in the *aox1a* or *ucp1* line as compared to their corresponding control in the presence of DTT in the high µM range. They also illustrate the sources of quantitative variability (i.e. absolute DTT concentration for the differential responses to arise; relative degree of inhibition in *aox1a* lines compared to the *ucp1* line).

It is difficult to realistically represent technical optimization and quality control of the setup suitably in the manuscript text, but we have done our best in the revision to make the properties of the setup as well as its limitations fully transparent.

A. We have now added an optimization experiment as new Suppl. Figure 2. The data illustrate the emergence of the genotypic differences at increasing DTT concentrations.

B. We explain at the start of the Results section that working with DTT as a plant treatment is inherently difficult to standardize due to the sensitivity of the thiols to oxidation. This is well recognized, e.g. in the ER stress community, but readers with any background need to be aware. In addition, we now specifically emphasize the experimental design that we used throughout the study to separate potential variability in effective DTT concentration from the differences in the responses of the genotypes:

"DTT is readily oxidized when dissolved in aqueous solutions, especially at elevated temperatures (Stevens et al., 1983), leading to variations of reduced, efficacious DTT in media plates between experiments. Hence, preparation of treatment media was strictly standardized (see Methods section) and comparisons between genotypes were performed through side-by-side comparisons on the same media plates. To consider the potential variability in effective concentration of the treatment DTT concentrations between 400 and 800 µM were routinely assessed (Figure 1, Supplemental Figure 2). Despite remaining variability in the overall effectiveness of the DTT treatment between experiments, the aox1a phenotype was strictly reproducible, as apparent by stronger impairment of root growth in the aox1a lines compared to their corresponding WT control in the presence of DTT in the high µM range. No obvious differential behavior was observed in green tissues between the genetic backgrounds, which is why we focused our further quantitative assessment on roots."

C. What remains is that the respective DTT concentration threshold for induction of the phenotype is variable within the window between <400 and >800 µM DTT. This is pointed out by the reviewer, by referring to the absolute differences in root length between the experiments. We would prefer to keep those data since they illustrate the technical variability between separate experiments, while they also show the robustness of the phenotype at the qualitative level. This information is important for future independent replication by other researchers.

2. I am not convinced that DTT is an effective respiratory substrate per se, although it clearly affects components of the mETC. The oxygen electrode data in Figure 6 are not convincing, especially the responses to respiratory inhibitors. I have added DTT to isolated mitochondria in many such experiments over the years and have not seen significant direct effects on oxygen uptake by mitochondria. This makes it unlikely, in my opinion, that reduced thiols will themselves act as respiratory substrates in vivo; rather, as the authors discuss, they are likely to affect respiration via NAD(P)H levels in the cytosol (in addition to stimulating AOX synthesis and activation). This could be tested using the publicly available ndb2 Arabidopsis KO mutants, since NDB2 is the major external NADH dehydrogenase in mitochondria. I think it is premature to say that reduced thiols act as respiratory substrates, and this statement in the abstract should be omitted.

We are grateful for the comprehensive feedback. First of all, it helps us to remove ambiguity that we accidentally introduced into our manuscript. We did not mean conclude that DTT acts as a direct respiratory substrate (like succinate or NADH) to the mETC. In other words, we did not mean to imply that any electron transport chain complex or protein of the mitochondrial inner membrane is able to oxidize DTT. Thanks to the comment by the reviewer we have now systematically checked the manuscript text, including the abstract, to remove statements that make that claim or are unnecessarily suggestive.

What we did mean to say, by sticking closely to the data, is that the electrons from the DTT thiols can make their way into the respiratory chain and drive proton pumping. This is an observation that we have been also surprised about. At the same time, it has intrigued us for several years, which is why we devised three different experimental approaches to thoroughly scrutinize it. Since we shared the skepticism of the reviewer about the oxygen-electrode based data and considered them potentially prone to passive oxidation artefacts, we established the assays for the PMF constituents, ΔpH and Δψ, which we believe provide a definitive case. The data that we include in the

manuscript represent a subset of the evidence-base that we generated, but the effects are coherent and reproducible. To illustrate both, coherence of the overall effect of DTT on ΔpH, as well as the variability between experiments, we have now added Suppl. Figure 9. Further we now emphasize more clearly that the oxygen electrode data on their own need to be inspected with caution, due to the low rates and the risk of artefacts. We have kept the oxygen consumption data in the manuscript since the effects were reproducible, and provided the starting point for the in-depth analysis of the PMF constituents by orthogonal approaches. While any single one of the three approaches employed has specific limitations, we find it hard to regard the evidence that all three combined provide anything other than compelling.

We find it difficult to argue with the point that the reviewer makes on their experience with DTT and isolated mitochondria. Maybe we can add that we initially shared their reservations, especially about the oxygen electrode data, and therefore put the data out for discussion within the plant mitochondrial community at the International Conference of Plant Mitochondrial Biology 2019 in Israel to obtain scrutiny. With respect to oxygen electrode-based measurements we received the feedback from two colleagues that DTT addition in the context of AOX activation resulted in minor 'drifts' in oxygen consumption in their hands, but those were small compared to those induced by the used respiratory substrates (which is consistent with our observations) and were dismissed as potential artefacts of passive DTT oxidation by oxygen without contribution to respiratory electron transport. Based on the personal exchanges and our best attempts to systematically consult the literature we could not obtain any information on previous attempts to delineate the effect of small thiols as net electron donors (direct or indirect) for the mETC of plant mitochondria.

We cannot currently pinpoint the exact route(s) of electron flux and the mechanisms/players by which the electron flux becomes sufficiently large for the observed effects. In the Discussion section we provide a selection of four plausible mechanisms, which are supported by the literature and the available data.

We agree with the reviewer that a mechanism of electron flux from DTT to the mETC via NAD is particularly attractive. We have now followed up on this possibility experimentally further using in vivo NAD redox measurement in the cytosol in response to acute DTT exposure of Arabidopsis seedlings. The new data are added as new Figure 5 and Suppl. Figure 6. Most interestingly NAD reduction in response to DTT was more pronounced in the absence of AOX1a, indicating that a bottleneck occurs for NADH oxidation. In a speculative model of electron flux via the NAD pool the question about the mechanism shifts to the mode of interaction between DTT and NAD, which deserves future investigation, and we are currently working towards getting a better picture (see our 2nd response to reviewer 1).

The reviewer suggests using NDB2 to test the hypothesis further. So far, our in vivo NADH/NAD^+ data confirm the concept of very efficient redox coupling of the NAD pools of the cytosol and the mitochondrial matrix as mediated by metabolite shuttles. As such net oxidation of the cytosolic NAD pool may also be achieved via matrix-exposed mETC dehydrogenases like Complex I and the NDAs, for which we have started selecting suitable genetic lines. Dissecting the relationship of the different NADH dehydrogenases in the context of reductive stress with all required controls will be an exciting endeavor and is likely to constitute an entire follow up project.

3. I think it should be pointed out that DTT not only induces AOX synthesis (or at least transcripts) but that it is also likely to ensure its activation by reducing the conserved cys-residues of the enzyme. In this context, I would like to see some western blots of AOX in root or leaf extracts with and without added reducing agents.

We have performed Western blotting using NEM-blocking of protein extracts from Arabidopsis seedling roots treated with mock, DTT or H_2O_2, followed by non-reducing PAGE and AOX detection by a polyclonal AOX1/2 antibody. The data are now included in Suppl. Figure 10.

They indicate that AOX1a is fully reduced in the root in vivo and DTT is unable to cause any further shift. This is in line with the concept that AOX proteins are typically not redox regulated in vegetative tissues under most conditions in vivo. Instead their cysteine thiols appear maintained in a reduced state in vivo, which is the prerequisite for regulation by organic acids. In that respect the basis of the argument of the reviewer may deviate from our understanding of the current evidence base. We would be excited to discuss this further together if there is a chance in the future. Like several other colleagues, we have recently tried to synthesize the available evidence into a picture on the question of AOX redox regulation in vivo. Here we would like to refer to a recent review (Meyer et al., 2021, Biol Chem):

"Despite the finding that reduction of the intermolecular disulfide of AOX dimers is required for full activity in

isolated mitochondria, the key question of whether the two Cys thiols represent a redox switch that is indeed operated in vivo had remained a matter of debate (Del-Saz et al. 2018; Millenaar and Lambers 2003; Riemer et al. 2015). For redox regulation to occur the Cys thiol will need to be oxidized preferentially over other Cys in the mitochondrial matrix, requiring either (i) high reactivity directly with a reactive oxygen species oxidant, like H2O2, or (ii) secondary oxidation via a specific thiol redox relay, as mediated by an oxidized TRX or a thiol peroxidase. Immunoblotting analyses of whole tissue extracts from different species (using suitable blocking of thiols to prevent oxidation after tissue disruption) have indicated that AOX is not oxidized in vivo, even under stress conditions. By contrast, AOX proteins are strongly oxidized in isolated plant mitochondria. Hence, AOX cysteines may passively oxidize during the organelle isolation process when the normal electron flows from metabolism into the thiol redox system ceases to provide a sufficiently negative redox potential (Umbach and Siedow 1997). A recent study has tested the impact of the absence of TRX-o1 on AOX1a redox status in A. thaliana (Florez-Sarasa et al. 2019). Despite TRX-o1 being the most likely TRX to reduce AOX1a, AOX1a redox status was unaffected, suggesting that efficient backup exists from other components of the matrix Cys redox system to keep AOX reduced (Schwarzländer and Fuchs 2019). This observation strongly suggests that AOX cannot be easily oxidized in vivo."

In addition to the Western blotting data we have followed the request of the reviewer and added a comment on the issue of AOX redox regulation and the potential for direct DTT-mediated activation in the Discussion section.

"The DTT effect in the absence of AOX1a is distinct from the stimulating effects that DTT has on AOX capacity in isolated mitochondria. There DTT is used to reduce an intermolecular disulfide bridge that covalently links AOX dimers. Yet, DTT exposure of seedling roots did not impact on the in vivo redox status of AOX1a of WT seedling roots in our experimental system (Supplemental Figure 10D,E). Instead, AOX1a was maintained in a fully reduced state, which is consistent previous in vivo observations (Millenaar and Lambers 2003; Del-Saz et al. 2018; Meyer et al., 2021)."

Reviewer #3 (Comments for the Author)

The manuscript by Fuchs et al provides evidence for a role of mitochondrial respiration to safeguard the ER from reductive stress. The manuscript is highly interesting, especially the possibility of alternative reductants feeding into mitochondrial respiration, but also in its use of novel biosensors, and has potential to attribute new function to mitochondrial stress responses. I have however a few concerns:

Major revisions:

General comment:

DTT has a very high reductive potential and therefore will have major impact on thiol balance, protein function/structure and other cellular components, i.e. it likely has a substantial pleiotropic effect. Although I agree that it can be used to identify genes/proteins involved in the response to such stress for further characterisation, as has been done for the UPRER, I find it much harder to attribute physiological responses to a specific process here. In my opinion the authors do not account sufficiently for such pleiotropic effects, e.g. by metabolic or transcriptomic experiments, to establish the extent of changes on a more global scale. Even a few targeted measurements, e.g. the concentrations/ratios of GSH/GSSG, ascorbate/dehydroascorbate and a few stress-markers by qRT-PCRs would shed more light on this. This would establish a baseline to interpret results on mitochondria and the ER more carefully. The authors rather reduce the DTT response to mitochondria-ER interactions without accounting for independent influences from other compartments, i.e. cytosol or chloroplasts, that may run in parallel.

We agree with the reviewer that the DTT treatment used in this study is likely to have broader effects. Following their specific suggestions, we have attempted to quantify GSH, GSSG, ascorbate, dehydroascorbate, as well as NAD^+, NADH, $NADP^+$ and NADPH in whole tissue extracts. Those measurements, however, rely on the redox-based colorimetric detection via MTT or NBT, which was heavily affected by the presence of DTT (even traces that remain present in the DTT treated samples after washing). To circumvent this technical problem, and in order to resolve the subcellular impact of DTT as suggested by the reviewer, we assessed the glutathione redox potential in response to DTT. We used different Arabidopsis lines that express the glutathione redox biosensor (roGFP2) in different subcellular compartments, i.e. the cytosol, the peroxisomes, the plastid stroma, the mitochondrial matrix and the ER lumen. This allows for a direct comparison of (a) the physiological glutathione redox poise of a given compartment and (b) the impact of DTT on shifting glutathione redox poise in a compartment. We have included the

new dataset as Suppl. Figure 3A. The data show that the ER is most oxidized of the five selected compartments under physiological conditions. It is therefore also most responsive to DTT. By contrast the Cys-based redox poise in the cytosol is already highly reduced under physiological conditions, meaning that DTT treatment is unable to cause any pronounced further shifts.

Given that DTT interacts with the cellular environment through the defined chemical reaction mechanism of thiol-disulfide exchange the data provide an estimate of the severity of the impact of DTT. While only few disulfides and other forms of oxidized Cys are present to be reduced by DTT in the cytosol, the peroxisomes, the mitochondrial matrix or the plastid stroma, oxidized Cys are highly abundant in the secretory system and need to be actively generated at very high rate in the ER. As such a much more pronounced quantitative impact of DTT in the ER is evident. That said other compartments will clearly also be affected to some extent either directly through reduction of specific oxidized Cys or indirectly, as a consequence of dysfunction of the secretory pathway. We have added a statement to the second paragraph of the Results section to explain the new dataset on the subcellular responses.

L144-148

Although I understand that DTT is somewhat difficult to work with, it is unclear to me how the apparent variable effect of DTT has been dealt with across experiment replicates. This somewhat lends itself to picking favorable outcomes, while discarding the unexpected ones, as an optimisation strategy? The differences in phenotypes of plants in Figure 1 and Suppl. Figure 3 for e.g. 800μM are quite substantial and suggest a two-fold variation of DTT can lead to the same result. I would assume that using fresh solutions each time would ameliorate these problems. Can the authors provide some clarification on this? Also, in some experiments different concentrations or submergence (up to 10mM DTT) have been used, which makes the comparison across experiments difficult.

We understand the concern. First, we need to politely but firmly reject the implication of potential cherry picking of results. Instead the intrinsic chemical characteristics of DTT for plant culture experiments has been a prime technical focus throughout this study, and the care that has been taken to guarantee robustness of the data goes several steps beyond preparing fresh DTT solutions, as we detail in the Methods section. We are very grateful for this critique, however. It demonstrates that we had failed to explain the measures we have taken to guarantee validity of the data and interpretations. In the revision we have clarified the issue by including additional data as Suppl. Figure 2 as well as textual revision. We would like to politely refer the reviewer also to our first response to reviewer #1 for additional details.

The reviewer points out the different DTT concentrations that were used for specific experiments. Those differences were selected based on the different nature of the experiments, i.e. long-term growth experiments on lower DTT concentrations, vs. acute response assays. The same rationale was followed with the use of tunicamycin and is based on common practice in the ER stress literature. The choice of DTT concentrations is now explained more clearly, and an optimization experiment for acute NAD reduction is added as Suppl. Figure 6.

L148:

The authors claim their DTT treatment has no phenotypic effect on leaf tissues, but provide no evidence for this. Again, in Suppl. Figure 7 the leaves look much bigger than for seedlings in Figure 1. Also, in leaf disc assays (Figure 5) the authors do find an effect of DTT treatment on green tissues. Some evidence should be presented that DTT does not have a general effect that impacts photosynthesis with a knock-on effect on (root) growth.

We are grateful to the reviewer for pointing out this shortcoming. What we were meaning to say was that there was a root growth phenotype for the comparison WT vs. *aox1a* or WT vs. *ucp1* in the presence of DTT, while we did not observe any obvious differences for this comparison with respect to their leaves. This was meant in the context of the antimycin A control, that does result in pronounced differences in leaf sizes between WT and *aox1a* seedlings (Figure 1C).

We have now rephrased the statement in the first section of the Results part to avoid any overinterpretation of the phenotyping data.

"No obvious differential behavior was observed in green tissues between the genetic backgrounds, which was not specifically investigated here. Instead we focused our further quantitative assessment on roots."

The reviewer points out that Figure 5 presented evidence for the impact of DTT treatment on NAD redox state using

leaf discs. We agree with the reviewer that developmental stage of the plants is ideally matched between experiments, which is why we have now repeated those experiments in young seedlings and expanded on the analysis by including the different genotypes. The new data are shown in the Figure 5 and Suppl. Figure 6. The effects are consistent with those observed before in leaf discs.

To address the question of potential effects of photosynthesis we have performed the DTT experiments while cultivating the seedlings in the dark. Here the problem arises that root growth is generally arrested even in the WT and does not allow to assess differential behavior between the genotypes. Since root growth arrest is a well-established result of light signaling we also used constant light exposure with the rationale that increased periods of photosynthetic activity. Here the total root growth was decreased in all lines but the differential root growth between genotypes was maintained. Those assays could clearly be expanded to usage of photosynthetic inhibitors etc – however even in the case that there was an impact of photosynthesis on the differential root growth (for which there is no indication), this would not impact on the key conclusions of this work.

L183:

The authors provide no further information for the ero1 ero2 line. It is therefore impossible to assess any results presented for this line. The authors need to decide to either remove this line from the manuscript or provide the necessary information on its generation, confirmation, characterisation etc.

We apologize that we were not more specific. We have added an advanced draft of a separate manuscript to our resubmission that describes and characterizes the *ero* lines in detail. This manuscript is about to be finalized and we will guarantee that it will be available as a preprint before this work will be published.

L203/211-212:

Is there any reason that the ucp1 line has this substantial variation and this is reproducible? This goes back to my earlier point on the selective inclusion of results based on phenotype (L144-148, see above). Also, as only two DTT concentrations are given it is hard to deduce 'optima'.

The larger biological variability of the *ucp1* line that the reviewer refers to in Figure 3 at 400 µM DTT was not systematically observed. A representative image of the data from that experiment is displayed in the same figure; the variability is real and this is a real experiment. Having inspected to raw data the effect arises since some individual show complete arrest of root growth.

‘Selective inclusion of results based on phenotype’ is an incorrect interpretation of the procedure that we followed and - yet again – we have to politely, but firmly reject the implication. There is an important difference between (a) effectiveness of the DTT treatment overall (as a prerequisite for differential growth between genotypes), and (b) the actual differential root growth effects between WT and mutant lines. To provide a better overview of both (a) and (b), we have added an exemplary DTT titration dataset as a new Suppl. Figure 2. Further data are provided as part of our first response to reviewer #1. The data clearly show the strict reproducibility of the phenotype at the qualitative level, i.e. stronger impairment of root growth in the *aox1a* or *ucp1* line as compared to their corresponding control in the presence of DTT in the high µM range. They also illustrate the sources of quantitative variability (i.e. absolute DTT concentration for the differential responses to arise; relative degree of inhibition in *aox1a* lines compared to the *ucp1* line).

L285-302

Although I like the use of biosensors, I feel direct measurement of respiration and including such data in a figure of the main manuscript is important. Although DTT is a tool, providing solid evidence for endogenous metabolites is crucial to support a physiological relevance. I know the latter is a somewhat stale argument, but I believe this is an important point for the story.

We share the feeling of the reviewer that direct and ‘classic’ respiratory measurements remain particularly meaningful in many circumstances. Their key advantage over the biosensing setup is that they can measure real electron fluxes. This is why we started the assays to dissect the relationship between DTT and the mETC using oxygen electrode measurements (as shown in Figure 6A-C; Suppl. Figure 7). We then complement those observations by the biosensor-based assays, however, to address the potential issue of oxygen consumption by passive DTT oxidation. Here the biosensor approach comes with the clear advantage that the PMF, as downstream

effect of mETC activity, can be measured.

With respect to endogenous metabolites, glutathione is clearly the prime candidate in this context since it is present in the cytosol and the mitochondria in vivo at mM concentrations and is the dominating endogenous small molecular thiol. Glutathione was assessed in its properties as electron source for mETC activity in Suppl. Figure 8A,C,D,E (we included a further dataset in Suppl. Figure 9F). The data show that the thiol can donate electrons that are efficiently passed to the mETC to run respiration in isolated mitochondria. For a further discussion of the potential in vivo role of endogenous GSH oxidation involving the mETC we would like to refer the reviewer to our 2nd response to reviewer #1. We also cover this point in the Discussion section.

L304-324

Although presented results clearly show that ANAC017 is involved in the stress response, I am not certain if the authors can ensure this is not a response to a pleiotropic effect unrelated to the ER. A comparison with TM treatment would allow to better differentiate. This might also show if mitochondrial stress impacts the ER, or if the ER stress impacts mitochondrial signalling.

Our working model cannot delineate where and how exactly ANAC017 signaling is initiated. Hence, we limit our speculative thoughts on that matter to the Discussion section. Instead our data suggest (a) that ANAC017 signaling is initiated in response to DTT and (b) that ANAC017 signaling is required for the mitochondria to adopt their protective role (as sufficiently accounted for by AOX1a induction).

The question of what upstream signal may initiate ANAC017 mechanistically is not a straightforward to address. This is a general limitation in our understanding and is also the case for the antimycin A model system, despite its extensive and highly successful usage. In the case of antimycin A the initial point of inhibition at Complex III is well defined, but the downstream effects of Complex III inhibition are extremely diverse (we documented reduction of NAD pool, depletion of ATP, increase of H_2O_2 production, accumulation of free Ca^{2+} in Wagner et al., 2020, New Phytol, but there are likely many more profound changes), so the connection between mitochondrial dysfunction and ANAC017 cleavage at the ER remains speculative, even though a ROS signal has been put forward as a plausible candidate. In analogy DTT has its major primary effect in the ER (see our first response to reviewer #3), but it is currently not realistic, nor the aim of this study, to delineate how exactly DTT expose leads to the activation of ANAC017 signaling.

The reviewer further suggests a comparison with tunicamycin (TM) treatments, as an independent ER stress inducer. We have used this treatment as a control for the experiments shown in Figure 7 and observed the same qualitative pattern as for DTT, i.e. inhibition in the *rao2.1* line and a trend towards inhibition in the *anac017-1* line, that did not reach significance, however (see dataset below). Those data provide some additional evidence in favor of ANAC017 activation as a consequence of ER stress. Yet, more independent experiments would be required to scrutinize such a model sufficiently. To avoid the danger of being overly suggestive we have not included the data in the manuscript, but would be happy to do so if the reviewer advises us to do so.

Primary root length increase of WT, rao2-1 and anac017-1 seedlings. (A) Seedlings were grown vertically on half-strength MS agar plates for 4 d, then transferred to fresh plates supplemented with the 250 ng mL-1 tunicamycin (TM). Comparison of primary root growth of WT with rao2.1 and anac017-1 mutant seedlings grown side-by-side. Representative images of seedlings 4 d after transfer. Scale bars: 10 mm. (B) Total root length 4 d after transfer on new plates. N = 12. Boxplot: 1st and 3rd quartiles with median and Tukey whiskers. Differences were tested after log-transformation of data to establish normal distribution and one-way ANOVA with Bonferroni's multiple comparisons test ($^{ns}P > 0.05$, $^{***}P < 0.001$).

Also, increased expression of AOX1a in an ire1a ire1b double mutant (Ng et al 2013), and increased expression of bZIP60 under AA treatment and in ANAC017 overexpressing lines (Meng et al 2019) already suggest a pathway from mitochondria to the ER. This is also missing from the discussion.

Thank you – we have incorporated both examples into the Discussion section.

Minor revisions:

The discussion is quite long (longer than the results) and could be more concise. But known connections between mitochondria and the ER, e.g. via ANAC017, bZIP60 (see above) should be discussed.

We agree and have now condensed the Discussion section (removed the most speculative section on hypoxia signalling) while adding critical points raised by the reviewers, including prior evidence on mechanisms to link mitochondrial and ER function.

L153-154:

Have the authors done concentration series for those stresses ? Otherwise this needs to be phrased more carefully, as other concentrations might have an effect.

Those treatments were applied in the form of a screening approach. While different concentrations of specific stressors were tested, such a screen cannot have the ambition to cover the full matrix of stimulus strengths comprehensively. The wording takes this into account when referring to the “other stress treatments that we tested”. However, we deleted the term ‘specific’ and provide a more quantitative statement on the DTT concentration (‘at exposure of seedlings to high µM concentrations of DTT’).

L548

Should be Supp Figure 6.

Thank you. Corrected. Further all Figure references have been updated.

I believe there is something wrong with the presentation of data. The scale is not centre around zero, I am unsure what the 0h timepoint would be calculated against, i.e. fold change against what?

Thank you for highlighting this lack of clarity in the representation of the data. The fold change in mRNA abundance is based on the average difference (expression intensities) of all probe sets for each condition in the two treatments (DTT and tunicamycin) (Martínez and Chrispeels, 2003). Since the data were presented in this manner in the original manuscript it seems appropriate to keep the presentation, but to provide the required clarity we have now added a statement to the legend of the figure, which is now Suppl. Figure 10.

Figure 2:

I am not sure if the time points in A and B should match, but I couldn't follow what was given in the legend, e.g. there is nothing marked in grey in A. Please check.

Thank you for making us aware that a part of the legend on panel B was already provided for panel A. Now corrected.

Suppl Figure 7 and 8: No reference anywhere in the text.

Thank you. Now all Suppl. Figures are referred to in the text. Following editorial instructions, it is now specified for all Supplemental Figures which main Figure they support.

TPC2021-RA-00408R1 2nd Editorial decision – *accept with minor revision* December 1, 2021

On the basis of the advice received, the board of reviewing editors would like to accept your manuscript for publication in The Plant Cell. This acceptance is contingent on revision based on the comments of our reviewers.

Reviewer #1 (Comments for the Author):

Reductive ER stress is a wide spread phenomenon with consequences for cell functioning. It is counteracted by a number of stress responses, but how these are coordinated at the physiological level is still investigated. The authors have set up an experimental system using DTT as reducing agent root growth in Arabidopsis as physiological readout. They provide evidence for a novel mechanism, at least for plants, where mitochondrial respiration acts a valve to release reducing power on the ER.

In this resubmission the authors provide a comprehensive rebuttal to all critical points raised. These are satisfying and in several instances now cleared by additional experiments. The authors explained why qPCR tests of expression of other AOX isoforms or UCP are not necessary. They elaborate at the question on how to determine electron fluxes through the glutathione redox systems and indirectly circumvent this problem by performing novel

cytosolic NAD redox measurements. The authors also responded with a new experiment to the request to independently test the hypothesis of alleviation of ER reducing stress via mitochondrial respiration providing and analyzing an aox1a-1 x ero1 double mutant. Input from yeast and mammalian investigations is also now integrated into the manuscript.

It may be considered to rephrase the title of the manuscript in a way that puts the hypothesis straight forward. As it is it reads as of quite specific and limited interest.

Considering the inherent difficulties of providing firm and mechanistic evidence for a physiologically operating network, I believe the authors now come close to final results having integrated the reviewers concerns.

Reviewer #2 (Comments for the Author):

The main points I raised have been dealt with satisfactorily. I suggest the following rewording of some of the headings and text:

Line 298

Mitochondrial electron transport chain activity is directly influenced by electrons derived from DTT

Line 326

Endogenous small thiol molecules can ALSO deliver electrons to the mitochondrial electron transport chain

Line 567

The finding that electrons from thiol oxidation can enter the respiratory chain, possibly by a mechanism that involves reducing NAD(P), expands.......

Reviewer #3 (Comments for the Author):

The authors have provided a comprehensive response to address my concerns and added additional data to support their revisions. I have only a few points to raise:

- In their reply the authors mention colorimetric methods for the quantification of GSH etc. I am no expert, but I am sure there are other methods, e.g.HPLC-based, to quantify these. However, I believe the biosensor-based experiments cover for this lack of direct measurements.

- L298: I would suggest to change the headline a bit. Maybe "DTT provides electrons to the mitochondrial electron transport chain" or similar to better fit what is described in this section, i.e. focus on DTT, and differentiates to the section that follows

- L439: please check the sentence for language

- For Supp Figure 10: I believe the fold variation is based on the comparison to the averaged controls ? I am still unsure why this color scale is not centred around 0.

- The authors largely discuss the use of thiol-derived electrons by the mETC under stress conditions, i.e. reductive stress. Can the authors comment if there is also scope for this under 'non-stress' conditions, i.e. a more general role for this electron source?

TPC2021-RA-00408R2 2nd Revision received December 16, 2021

Reviewer comments and **author responses**:

Reviewer #1 (Comments for the Author): Reductive ER stress is a wide spread phenomenon with consequences for cell functioning. It is counteracted by a number of stress responses, but how these are coordinated at the physiological level is still investigated. The authors have set up an experimental system using DTT as reducing agent root growth in Arabidopsis as physiological readout. They provide evidence for a novel mechanism, at least for plants, where mitochondrial respiration acts a valve to release reducing power on the ER. In this resubmission the

authors provide a comprehensive rebuttal to all critical points raised. These are satisfying and in several instances now cleared by additional experiments. The authors explained why qPCR tests of expression of other AOX isoforms or UCP are not necessary. They elaborate at the question on how to deter-mine electron fluxes through the glutathione redox systems and indirectly circumvent this problem by performing novel cytosolic NAD redox measurements. The authors also responded with a new experiment to the request to independently test the hypothesis of alleviation of ER reducing stress via mitochondrial respiration providing and analyzing an aox1a-1 x ero1 double mutant. Input from yeast and mammalian investigations is also now integrated into the manuscript. It may be considered to rephrase the title of the manuscript in a way that puts the hypothesis straight forward. As it is it reads as of quite specific and limited interest. Considering the inherent difficulties of providing firm and mechanistic evidence for a physiologically operating network, I believe the authors now come close to final results having integrated the reviewers concerns.

We thank the reviewer for assessing the revision in detail and feel glad that our explanations and edits address their previous suggestions and concerns. On potentially rephrasing the title, we understand the point that it is phrased to be specific and does not generalize the findings. We have been discussing different alternatives to broaden its scope but they all come with disadvantages, most importantly the danger of over-interpretation. Hence, we are open for modifications to the title and would welcome specific suggestions for modifications, from the editorial side or the Science Editors. For now, we have left the title unchanged though since it strikes a balance between scope and accurately representation of the findings.

Reviewer #2 (Comments for the Author): The main points I raised have been dealt with satisfactorily. I suggest the following re-wording of some of the headings and text: Line 298 Mitochondrial electron transport chain activity is directly influenced by electrons de-rived from DTT

Thank you. The same issue was raised by reviewer #3. Now corrected.

Line 326 Endogenous small thiol molecules can ALSO deliver electrons to the mitochondrial electron transport chain

Thank you. Corrected.

Line 567 The finding that electrons from thiol oxidation can enter the respiratory chain, possibly by a mechanism that involves reducing NAD(P), expands.......

Thank you. Corrected.

Reviewer #3 (Comments for the Author): The authors have provided a comprehensive response to address my concerns and added additional data to support their revisions. I have only a few points to raise:

- In their reply the authors mention colorimetric methods for the quantification of GSH etc. I am no expert, but I am sure there are other methods, e.g.HPLC-based, to quantify these. However, I believe the biosensor-based experiments cover for this lack of direct measurements.

The reviewer makes a fair point. We did not mean to say that there are no alternative methods. The approach that we decided to take comes with the advantage of differentiating between subcellular locations, and we are grateful that the reviewer appreciates the experimental strategy that we took to address their comment from the initial review round.

- L298: I would suggest to change the headline a bit. Maybe "DTT provides electrons to the mitochondrial electron transport chain" or similar to better fit what is described in this section, i.e. focus on DTT, and differentiates to the section that follows

Thank you. The same issue was raised by reviewer #2. Now corrected.

- L439: please check the sentence for language

Thank you. Now corrected to: "This phenotype did not occur in the absence of DTT in any of the experiments performed here. It is tempting to speculate, however, that the specific cultivation under which root growth impairment was observed may have induced a reductive challenge from an endogenous source."

- For Supp Figure 10: I believe the fold variation is based on the comparison to the averaged controls? I am still unsure why this color scale is not centred around 0.

Thank you. We have revised the false colour scale in the figure to be symmetrical and centred around 0 to improve clarity.

- The authors largely discuss the use of thiol-derived electrons by the mETC under stress conditions, i.e. reductive stress. Can the authors comment if there is also scope for this under 'non-stress' conditions, i.e. a more general role for this electron source?

Thank you. We had discussed our thoughts on this exciting question in the response to Reviewer #1 in the previous response letter. We have not gone as far in the Discussion section, since our thoughts are currently largely speculative, albeit deserving of further testing. The relevance of the metabolic link between thiols and respiration in non-stress scenarios is addressed in the Discussion section at the end of the paragraph on 'Mechanisms of interaction between respiratory metabolism and thiol redox regulation'. We now added the following:

"The extent to which the mETC may act as an electron sink to endogenous thiols, such as glutathione, also under non-stress conditions, and the rates at which such a mechanism may operate, deserve further investigation. Steady oxidation of glutathione by a mechanism that involves the mETC, as balanced by steady reduction by glutathione reductases at the expense of metabolically-derived NAD(P)H, may constitute an efficient mechanism to rapidly adjust glutathione redox potential. In such a hypothetical model the mitochondria would make up the downstream section of a pathway that delivers net 'glutathione oxidase' activity by hosting a steady flux of glutathione-derived electrons. Such a concept of glutathione redox control does not hinge of the presence of AOX and may be applicable in various eukaryotes also beyond plants."

**TPC2021-RA-00408R2 3rd Editorial decision – *acceptance pending***	**December 18, 2021**

We are pleased to inform you that your paper entitled "Reductive stress triggers ANAC017-mediated retrograde signaling to safeguard the endoplasmic reticulum by boosting mitochondrial respiratory capacity" has been accepted for publication in The Plant Cell, pending a final minor editorial review by journal staff. At this stage, your manuscript will be evaluated by a Science Editor with respect to its presentation of scientific content, compliance with journal policies, and presentation for a broad readership.

**Final acceptance from Science Editor**	**January 14, 2022**
